# Exosomes in early lung cancer diagnostics: the current state of progress made and prospects

**DOI:** 10.3389/fcell.2025.1739242

**Published:** 2026-01-07

**Authors:** Xiaotian Liu, Xianlin Xu, Qian Wang

**Affiliations:** 1 Department of Anesthesiology, Children’s Hospital of Soochow University, Suzhou, China; 2 Sinopharm Dongfeng General Hospital, Hubei University of Medicine, Shiyan, China

**Keywords:** early diagnosis, exosomal biomarker, exosome, liquid biopsy, lung cancer

## Abstract

The high mortality rate of lung cancer primarily results from its late-stage diagnosis, at which point metastasis has usually occurred and therapeutic options are limited, leading to an overall 5-year survival rate below 20% in most countries. The current screening method, low-dose computed tomography (LDCT), faces challenges such as high false-positive rates, which can result in overdiagnosis and unnecessary surgeries, as well as the risk of cancer due to repeated exposure to ionizing radiation. Although tumor tissue detection remains the gold standard for cancer diagnosis, it is limited by invasive sampling procedures that may cause patient trauma, as well as by tumor heterogeneity and inconsistent tissue quality, which can compromise diagnostic accuracy. Due to these challenges among others, researchers have been exploring better diagnostic methods that are not only sensitive and specific but also non-invasive, utilizing easily available samples with good reproducibility. In recent years, studies have revealed that humoral-derived materials, such as exosomal RNAs and proteins are considered the most promising biomarkers for the early diagnosis of lung cancer in body fluids owing to their stability, accessibility, and specificity. This study reviews current research on the exploration of exosomes as early diagnostic markers for lung cancer. Both established methods and emerging technologies, such as surface-enhanced Raman spectroscopy (SERS), lateral flow immunoassays (LFIA), microfluidics, and electric field-induced release and measurement (EFIRM), as well as commercial products, are discussed.

## 1 Introduction

According to global cancer statistics, lung cancer ranks as the most frequently diagnosed cancer (accounting for 12.4% of all cases) and the foremost cause of cancer-related mortality (responsible for 18.7% of all cancer deaths) when considering both sexes combined (Bray et al., 2024). Non-small cell lung cancer (NSCLC) represents the most prevalent form of lung cancer, encompassing two primary pathological subtypes: adenocarcinoma (AdCa) and squamous cell carcinoma (SCC). The 5-year relative survival rate for lung cancer remains below 20% in the majority of countries, underscoring the critical importance of early detection in reducing the disease’s mortality rates (Fox et al., 2023). Current screening methodologies, including low-dose computed tomography (LDCT), volatile organic compound detection, exhaled breath condensate analysis, and genomic profiling, each offer unique advantages and limitations (Fox et al., 2023). While LDCT is the predominant method for detecting NSCLC or pulmonary nodules, it carries the risk of overdiagnosis, potentially leading to unnecessary surgical interventions due to high false-positive rates (Hendrick and Smith, 2024). LDCT studies conducted in Europe have demonstrated less pronounced efficacy of this technique, leading to its non-recommendation for lung cancer screening. The limitations associated with early lung cancer screening include the elevated risk of cancer and increased costs due to repeated exposure to ionizing radiation (Grannis, 2022; Kerpel-Fronius, 2022). While additional blood-based assays have been developed to augment imaging techniques, the serum protein biomarkers currently employed for NSCLC—including carcinoembryonic antigen (CEA), cytokeratin 19 fragment (CYFRA21-1), and SCC antigen—continue to display inadequate diagnostic specificity and sensitivity (Huang et al., 2022; Saman et al., 2022). The pursuit of non-invasive, specific, and sensitive diagnostic methodologies for lung cancer has been the focus of extensive research. Numerous studies have indicated that, beyond serum and plasma, other bodily fluids such as sputum, saliva, and urine are promising samples for disease diagnosis through the analysis of their nucleic acid or protein constituents (Huang et al., 2022; Saman et al., 2022; Khan and Saraya, 2023; Skallevold et al., 2021).

Extracellular vesicles (EVs) constitute a heterogeneous group of membrane-bound particles released by cells, broadly classified by their biogenesis and physical characteristics. Exosomes, traditionally referred to as small EVs, have diameters ranging from approximately 30–150 nm, and are known to encapsulate lipids, proteins, and nucleic acids, including RNAs and DNAs (Xu X. et al., 2024). These exosomal cargoes are released into the microenvironment, serving as vehicles for intercellular communication. Within the tumor microenvironment, these molecular particles play a critical role in tumor pathogenesis and hold significant potential as diagnostic and prognostic biomarkers. For instance, microRNAs (miRNAs) such as let-7, miR-137, miR-182, miR-221, and miR-372, which are expressed in lung cancer, may serve as predictive markers for the survival rates of lung cancer patients (Lv and Xiong, 2024). In the context of early diagnosis, elevated levels of miR-205 and miR-375 can distinguish SCC from AdCa in lung cancer biopsies (Song et al., 2023). Furthermore, microarray data analysis has identified a miRNA expression profile comprising 32 miRNAs that effectively differentiates AdCa from SCC, with let-7e, miR-191, miR-25, miR-34a, and miR-34c demonstrating prognostic significance (Liu et al., 2023). Circulating miRNAs have been extensively investigated for their potential roles in noninvasive diagnostics (Fehlmann et al., 2020). For instance, the expression profiles of specific groups of miRNAs, comprising seven and nine miRNAs, have been identified as noninvasive biomarkers for the diagnosis and prognosis of AdCa and SCC, respectively (Niu et al., 2019).

Beyond these exosomal nucleic acids, the protein and lipid contents of exosomes have also been examined for their utility in the early diagnosis of lung cancer. This paper provides a comprehensive review of the current research landscape concerning the application of exosomal components as biomarkers for the early detection of lung cancer. It explores the involvement of exosomes in lung cancer pathogenesis, their potential as biomarker targets, and both established and emerging methodologies for exosome detection.

This narrative review was based on a structured literature search of PubMed, Scopus, and Web of Science conducted between January 2010 and March 2025. Studies were identified using combinations of the terms “lung cancer”, “exosome”, “early diagnosis”, “exosomal biomarker”, and “liquid biopsy” with Boolean operators. The reference lists of relevant articles were also examined to ensure comprehensive coverage. Eligible studies included original research and high-quality review articles focusing on the diagnostic potential of exosomal RNAs, proteins, and lipids in lung cancer, whereas conference abstracts, non-English publications, and studies lacking relevance to early detection were excluded. Both human and experimental studies were considered, and the evidence was synthesized qualitatively.

## 2 Contribution of exosomes to lung cancer development

**FIGURE 1:**
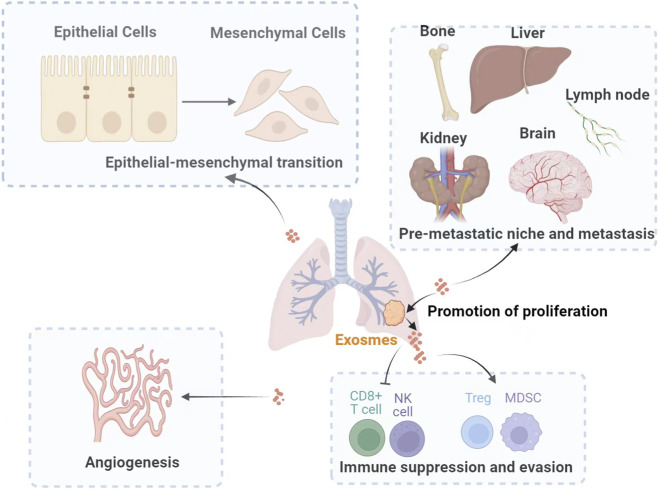
The mechanisms by which exosomes contribute to lung cancer onset and progression. Exosomes are integral to the initiation and progression of lung cancer through various mechanisms. They facilitate tumor cell proliferation, induce immune suppression and evasion, promote epithelial-mesenchymal transition, and establish a pre-metastatic niche conducive to metastasis. Furthermore, exosomes enhance angiogenesis, thereby accelerating tumor growth and dissemination (Treg: regulatory T-cells; MDSC: myeloid-derived suppressor cell).

It is well-established that exosomes facilitate the transfer of molecular constituents that remodel the tumor microenvironment, thereby contributing to carcinogenesis (Liu X. et al., 2024). These tumor-derived exosomes facilitate intercellular communication by transferring a variety of biomolecules, including chemokines, growth factors, RNAs, proteins, and lipids (Kewitz-Hempel et al., 2024; Castillo-Peña and Molina-Pinelo, 2023). For example, research indicates that extracellular vesicles circulating in individuals at high risk for lung cancer can induce a pro-tumorigenic transformation of stromal cells through the transfer of microRNAs such as miR-126 and miR-320. These microRNAs promote a pro-angiogenic phenotype in endothelial cells and M2 polarization in macrophages (Pontis et al., 2021). Exosomes contribute to the initiation and progression of lung cancer by promoting cell proliferation, immune evasion, epithelial–mesenchymal transition (EMT), pre-metastatic niche formation, and angiogenesis, as depicted in Figure 1.

### 2.1 Tumor cell proliferation

The proliferation of tumor cells, a critical phase in cancer progression, is reliant on soluble growth factors and other regulatory molecules delivered by exosomes. Consequently, exosomes play an active role in modulating the tumor microenvironment and influencing tumor cell proliferation and progression. Long non-coding RNA (LncRNA) UFC1 is upregulated in serum exosomes, tumor tissues, and serum of patients with NSCLC, and shows a positive correlation with tumor infiltration. Silencing UFC1 results in the inhibition of NSCLC cell proliferation, migration, and invasion, while inducing cell cycle arrest and apoptosis (Zang et al., 2020). This suggests that exosomes derived from NSCLC cells facilitate NSCLC cell proliferation, migration, and invasion through the transfer of regulatory molecules. Additionally, circSATB2 is highly expressed in NSCLC cells and tissues and positively regulates the expression of fascin homolog 1, actin-bundling protein 1 (FSCN1) via miR-326. Exosomes transfer circSATB2 to enhance the proliferation, migration, and invasion of NSCLC cells, and also induce abnormal proliferation in normal human bronchial epithelial cells (Zhang et al., 2020). In a separate investigation, researchers demonstrated that circ-CPA4 modulates cell proliferation, stemness, motility, and drug resistance in NSCLC cells. Additionally, it inactivates CD8^+^ T cells within the tumor microenvironment through the let-7 miRNA/programmed death-ligand 1 (PD-L1) axis (Hong et al., 2020). Furthermore, the overexpression of Wnt5b, a protein belonging to the same family as Wnt5a, has been linked to increased cancer aggressiveness. Wnt5b-associated exosomes are biologically active, promoting the proliferation and migration of A549 lung AdCa cells and enhancing Wnt5b signaling in Chinese hamster ovary (CHO) cells (Harada et al., 2017).

### 2.2 Immune suppression and evasion

Exosomes facilitate the transfer of immunosuppressive molecules either through paracrine signaling or direct interaction with immune cells, leading to the suppression of anti-tumor functions and the promotion of a tumor-supportive microenvironment. Consequently, tumor-derived exosomes modulate antitumor immune responses by delivering materials such as mRNA, miRNA, and DNA, which inhibit T-cell activation and proliferation, increase the presence of regulatory T-cells (Tregs) and myeloid-derived suppressor cells (MDSCs), and suppress the function of natural killer (NK) and CD8^+^ T-cells, thereby effectively evading the host immune system (Zandberg et al., 2024; Zabeti et al., 2024). Additionally, tumor cells evade immune surveillance by upregulating the surface expression of PD-L1, which interacts with programmed death-1 (PD-1) receptors on T cells to trigger the immune checkpoint response. Notably, tumor-derived exosomes express PD-L1 on their surface, which suppresses CD8 T cell function, thereby promoting tumor growth and influencing the outcome of anti-PD-1 therapy (Chatterjee et al., 2021). Furthermore, this mechanism is associated with the induction of systemic anti-tumor immunity and memory (Ayala-Mar et al., 2021). Exosomes are implicated in the induction of immune escape mechanisms in tumor cells, such as the evasion of immunogenic cell death through damage-associated molecular patterns (DAMPs) and the expression of surface recognition molecules including CD39/CD73, CD38, CD47, cytotoxic T-lymphocyte antigen 4 (CTLA-4, also known as CD152), lymphocyte-activation gene 3 (LAG-3), V-domain immunoglobulin suppressor of T cell activation (VISTA), and T cell immunoglobulin and mucin domain 3 (TIM-3) (Zhang et al., 2015; Wo et al., 2019). Furthermore, exosomes facilitate immune escape by delivering immunosuppressive molecules such as transforming growth factor beta (TGF-β), vascular endothelial growth factor (VEGF), interleukin 10 (IL-10), and prostaglandin E2 (PGE2) (Bojović et al., 2020; Mortezaee et al., 2023).

### 2.3 Epithelial-mesenchymal transition

EMT represents a complex phenotypic transformation that enhances cellular motility and invasiveness by disrupting extensive epithelial cell-cell interactions, apical-basal polarity, and distinct cytoskeletal structures. As a pivotal event in tumor metastasis, EMT is associated with alterations in exosomal protein and RNA expression, which contribute to the promotion of EMT, as well as the migration and invasion of cancer cells (Lv and Xiong, 2024). Cancer-associated fibroblasts (CAFs) play a pivotal role in tumor progression by transporting exosomes to adjacent cells. These exosomes, expressed by CAFs, contain cargoes such as miR-210, which can facilitate EMT by targeting UPF1 and activating the PTEN/PI3K/AKT signaling pathway, thereby enhancing the migration and invasion of NSCLC (Yang et al., 2020). Similarly, miRNA-92a promotes EMT through the activation of the PTEN/PI3K/AKT signaling pathway in NSCLC metastasis (Lu et al., 2017). Other studies have demonstrated that exosome cargo reflects the TGF-β1-mediated EMT status in human lung AdCa cells (Kim et al., 2016) and contributes to resistance against osimertinib in epidermal growth factor receptor (EGFR)-mutant NSCLC (Hisakane et al., 2021).

### 2.4 Pre-metastasis niche and metastasis

Lung cancer remains the leading cause of cancer-related mortality, accounting for 24% of all cancer deaths. Metastasis, a critical phase in lung cancer progression, is associated with over 70% of these fatalities. Cancer-derived exosomes are regarded as significant drivers of cancer-induced pre-metastatic niche formation at distant sites, as well as metastasis (Li et al., 2024). Hoshino and colleagues have demonstrated that tumor-derived exosomes, including those originating from the lungs of both mice and humans, are taken up by organ-specific cells such as lung fibroblasts, epithelial cells, liver Kupffer cells, and brain endothelial cells, thereby facilitating the preparation of the pre-metastatic niche. Furthermore, treatment with exosomes from lung-tropic models was found to redirect the metastasis of bone-tropic tumor cells, with exosomal integrins α6β4 and α6β1 being associated with lung metastasis (Hoshino et al., 2015). Exosomal RNAs also play a significant role in the pre-metastatic and metastatic stages of lung cancer. For instance, circSATB2 is markedly upregulated in serum exosomes from lung cancer patients, demonstrating high sensitivity and specificity for clinical detection and being linked to lung cancer metastasis (Zhang et al., 2020).

### 2.5 Angiogenesis

Angiogenesis refers to the formation of new blood vessels from existing ones. Exosomes contribute to the angiogenic process in cancer progression by transporting various pro-angiogenic biomolecules, such as VEGF, matrix metalloproteinases (MMPs), and microRNAs, while also downregulating factors that inhibit hypoxia-inducible factor 1 (HIF-1) (Olejarz et al., 2020). Within the tumor microenvironment, the uptake of exosomes by normal endothelial cells activates angiogenic signaling pathways, leading to the formation of new blood vessels. Cancer-derived exosomal miR-25-3p facilitates the establishment of a pre-metastatic niche by enhancing vascular permeability and promoting angiogenesis (Zeng et al., 2018). The overexpression of miR-210 further augments angiogenesis, as it is transferred via exosomes derived from lung cancer cells, functioning as a proangiogenic factor in cancer-associated fibroblasts through modulation of the JAK2/STAT3 pathway (Fan et al., 2020). Additionally, miR-23a is markedly elevated in exosomes originating from lung cancer, directly targeting and suppressing prolyl hydroxylase 1 and 2 (PHD1 and 2) and inhibiting the tight junction protein ZO-1. This suppression leads to the accumulation of HIF-1α in endothelial cells, thereby enhancing angiogenesis under both normoxic and hypoxic conditions, and increasing vascular permeability and cancer transendothelial migration (Huang et al., 2024).

## 3 Heterogeneity of exosomes in theranostic applications

Exosomes offer unique advantages in theranostics due to their endogeneity and heterogeneity, surpassing traditional synthetic carriers like liposomes and nanoparticles (Pegtel and Gould, 2019). Their molecular heterogeneity presents both challenges and opportunities in lung cancer biomarker development, as exosomes consist of diverse subpopulations with different cellular origins and molecular contents. This diversity is influenced by the parent cell’s pathophysiological state, allowing tumor-derived exosomes to carry specific molecular signatures indicative of the cancer’s genotype and phenotype (Rahimian et al., 2024; Hanjani et al., 2022). In NSCLC, exosomal proteins such as EGFR and PD-L1 reflect the tumor’s mutational status and immunosuppressive properties (Jakobsen et al., 2015). Additionally, exosomal RNA cargo, including microRNAs like miR-21 and miR-4257, exhibit distinct profiles that can distinguish lung AdCa from SCC and benign pulmonary conditions (Zhu et al., 2025). This heterogeneity serves as a valuable resource for the identification of highly specific and sensitive biomarkers, advancing beyond single-molecule analytes to encompass complex, multi-analyte signatures for early detection, subtyping, and prognostic stratification. Nonetheless, this same heterogeneity poses significant challenges for the analytical and clinical validation of exosomal biomarkers, as elaborated upon later in this manuscript.

The yield, purity, and molecular cargo of exosomes are strongly influenced by pre-analytical and analytical variables, which represent a major source of technical variability in biomarker studies (Welsh et al., 2024; Verma and Arora, 2025). Key collection-related factors include the choice of anticoagulant, donor fasting status, and the time interval between blood draw and sample processing, all of which can affect hemolysis, particle concentration, and EV purity. Sample processing parameters, including centrifugation speed, duration, rotor type, number of spin cycles, and exposure to repeated freeze-thaw cycles, have been shown to alter EV yield, integrity, and subtype composition, thereby affecting the reproducibility of downstream proteomic and nucleic acid analyses (Welsh et al., 2024; Verma and Arora, 2025). In addition, the choice of isolation method has a substantial impact on recovery and cargo composition. Differential ultracentrifugation, polymer-based precipitation, size-exclusion chromatography (SEC), immunoaffinity capture, and emerging microfluidic platforms introduce different trade-offs between yield, purity, and enrichment of specific EV subpopulations, which can influence biomarker performance (Welsh et al., 2024; Verma and Arora, 2025). To promote transparency and reproducibility, studies should adhere to the Minimal Information for Studies of Extracellular Vesicles (MISEV2023) guidelines and report essential methodological metadata, including sample volume, type of collection tube and anticoagulant, donor fasting status, time to processing, centrifugation conditions, storage parameters, number of freeze-thaw cycles, detailed isolation protocols, particle counts per milliliter, protein concentration, EV marker validation, and normalization strategies for downstream analyses (Welsh et al., 2024). Standardized reporting of these parameters is critical for inter-study comparability and for distinguishing true biological variation from technical artifacts.

## 4 Sources of samples for exosomes in lung cancer diagnosis

Exosomes can be isolated from a variety of biofluids — both blood and non–blood sources — in cancer diagnostics. In the context of lung cancer, studies have already demonstrated that exosome-derived miRNAs can be recovered from peripheral blood (plasma/serum), saliva, urine, sputum, as well as lung-specific fluids like bronchoalveolar lavage fluid (BALF) and pleural effusion or pleural lavage (Verma et al., 2025). The availability of exosomes in these diverse biofluids reflects the fact that tumor-derived vesicles are shed into both the circulatory system and local lung microenvironments; thus, sampling may occur through standard blood draws or more lung-targeted procedures (e.g., BAL, pleural fluid aspiration), depending on clinical context and invasiveness accepted (Verma et al., 2025; Martínez-Espinosa et al., 2024).

### 4.1 Blood-derived exosomes in lung cancer diagnosis

The concept of blood-based biomarkers is well-established. For several years, tumor protein biomarkers such as CEA, alpha-fetoprotein (AFP), and carbohydrate antigen 19–9 (CA19-9) have been extensively utilized in the screening and diagnosis of lung cancer, evaluation of treatment efficacy, postoperative monitoring, and prognostic assessment (Wojtalewicz et al., 2023; Bitter et al., 2023). Despite their utility in clinical practice, certain biomarkers lack specificity and may be upregulated in non-tumor conditions. Fortunately, alternative blood-based biomarkers, such as exosomes and their cargo, have demonstrated promising diagnostic accuracy, characterized by high sensitivity, specificity, reliability, and reproducibility for lung cancer. Consequently, these biomarkers continue to be actively investigated. Exosomes released into circulation possess the potential to provide insights into tumor status, as evidenced by the correlation between plasma exosomal microRNAs and the efficacy of immunotherapy in EGFR/ALK wild-type advanced NSCLC (Liu F. et al., 2024). Additionally, plasma exosomal proteins, including SRGN, TPM3, THBS1, and HUWE1, have been shown to differentiate lung AdCa from normal controls (Vykoukal et al., 2017).

However, tumor-derived exosomes represent only a small fraction of the total exosome population in blood, and the presence of abundant lipoproteins and platelets complicates the isolation of pure exosomes. To address these challenges, MISEV2023 guidelines recommend that studies report all relevant experimental conditions clearly, including anticoagulant type, centrifugation protocols, and methods used to isolate exosomes, ensuring that co-isolates like platelets and lipoproteins are effectively removed (Welsh et al., 2024). Despite many promising findings (e.g., protein or miRNA markers), most studies remain relatively small or single-center, and large-scale validation across diverse populations is still lacking.

### 4.2 Saliva-derived exosomes in lung cancer diagnosis

Exosomes, along with other microvesicles, have been successfully isolated from human saliva and subjected to comprehensive characterization. These salivary extracellular vesicles contain informative proteins, RNAs, and DNAs that serve as potential biomarkers for the screening and early detection of lung cancer. Exosomes function as protective carriers for tumor cell-specific mRNAs and proteins in body fluids, potentially facilitating their transmission from blood to saliva. This suggests a link between distal tumor progression and biomarker discovery in saliva (Cui et al., 2024). Ultra-short circulating tumor DNA (usctDNA) has been predominantly localized within the exosomal fraction of saliva and plasma in patients with NSCLC, highlighting its potential as a novel target for liquid biopsy in lung cancer detection (Wang F. et al., 2024). One study successfully identified and quantified 785 proteins from salivary exosomes and 910 proteins from microvesicles, with 150 and 243 proteins, respectively, being identified as dysregulated candidates in the context of lung cancer (Sun et al., 2018). In a similar proteomic profiling study using liquid chromatography-mass spectrometry/mass spectrometry (LC-MS/MS) on saliva and serum exosomes from lung cancer patients, researchers identified 319 and 994 exosomal proteins, respectively, and discovered 11 potential lung cancer-specific candidates present in both body fluids (Sun et al., 2017).

Although saliva offers a non-invasive, easy-to-collect biofluid, its complexity poses challenges. Salivary exosomes contain proteins, RNAs, and DNAs that can serve as potential biomarkers. However, the complexity of saliva, which contains enzymes, antibodies, and microbial products, can obscure the detection of tumor-derived exosomes. Saliva flow rate, oral hygiene, food intake, and microbial contamination can all influence exosome isolation and cargo analysis. To minimize variability, studies should standardize collection protocols, assess the impact of food intake on saliva composition, and follow MISEV guidelines to ensure accurate reporting of collection methods and processing conditions (Welsh et al., 2024). To date, reports of salivary exosomal biomarkers for lung cancer remain scarce, and any findings should be viewed as preliminary until validated in larger, multi-center studies.

### 4.3 Urine-derived exosomes in lung cancer diagnosis

Urine, as one of the most accessible biofluids, has been increasingly utilized in clinical diagnostics and biomedical research. Over the past decade, urinary extracellular vesicles have been shown to reflect molecular processes and physiological and pathological conditions across various tissues. Consequently, several methods have been developed to isolate and characterize these vesicles to explore their clinical applications (Erdbrügger et al., 2021). Recent advancements in analytical methodologies, particularly those utilizing mass spectrometry (MS)-based proteomic analysis, have facilitated high-throughput and comprehensive proteome profiling of urinary extracellular vesicles. This has significantly contributed to the discovery, quantification, and characterization of cancer-specific exosome biomarkers. Urinary exosomes are emerging as a valuable source of biomarkers not only for lung cancer but also for bladder, prostate, kidney, esophageal, and colorectal cancers (Wang X. et al., 2024). A novel integrated microfluidic device has been developed specifically for the isolation and *in situ* detection of lung cancer-specific exosomes from patient urine samples. This device boasts a detection limit of fewer than 1,000 exosome particles per milliliter and has been validated using 500 μL urine samples from both lung cancer patients and control subjects. It demonstrates a promising capability to differentiate early-stage lung cancer patients from healthy individuals (Di Bella and Taverna, 2024).

Urine offers a completely non-invasive and easily repeatable source of exosomes, but in the context of lung cancer, its use faces several constraints. First, exosome yield and concentration in urine may be low, since vesicles must pass through renal filtration or derive from urinary tract cells rather than lung tissue, which reduces the “tumor fraction” — making lung-tumor–specific signals likely very diluted and potentially masked by exosomes from kidney or bladder cells (Martínez-Espinosa et al., 2024). Additionally, urine’s variable matrix (pH, osmolarity, presence of salts, urea, metabolites) may degrade or alter exosome integrity or cargo stability, complicating reproducible isolation (Martínez-Espinosa et al., 2024). Given the variability in urinary exosome levels, normalization strategies (e.g., urinary creatinine or total protein concentration) should be employed, as recommended by MISEV2023, to account for these differences (Welsh et al., 2024). Hence, while urinary exosomes are attractive for noninvasive diagnostics, evidence for lung cancer-specific markers in urine remains limited and requires rigorous validation in larger, well-controlled cohorts.

### 4.4 Sputum-derived exosomes in lung cancer diagnosis

Over the past six decades, sputum cytology has been a fundamental component of lung cancer screening, complementing other diagnostic techniques such as chest X-ray, computed tomography (CT), fluorescence endoscopy, and LDCT (Adams et al., 2023). Recent research on pulmonary diseases and cancer has identified the presence of extracellular vesicles in airway secretions, including sputum, bronchoalveolar lavage fluid (BALF), nasal lavage (NL), and pharyngeal lavage (He et al., 2024). Given the pivotal role of extracellular vesicles from these secretions in the pathophysiology of pulmonary conditions, they are considered potential biomarkers. Substantial epidemiological evidence supports the association between idiopathic pulmonary fibrosis and lung cancer (Tzouvelekis et al., 2019). Exosomes isolated from the sputum of patients with idiopathic pulmonary fibrosis exhibited a significantly elevated level of exosomal miR-142-3p (8.06-fold increase, p < 0.0001), which was positively correlated with the percentage of macrophages in the sputum (Guiot et al., 2020). A related study similarly identified significant dysregulation in sputum exosomal miRNA levels between patients with lung fibrosis and healthy individuals, revealing a distinct miRNA signature (miR-142-3p, miR-33a-5p, let-7d-5p) that differentiates patients from healthy controls with high sensitivity and specificity. Notably, miR-142-3p and let-7d-5p are recognized for their roles in EMT, a critical process in lung tumorigenesis, while miR-33a-5p has been associated with liver fibrosis (Njock et al., 2019). These findings suggest the potential utility of sputum-derived exosomes as biomarkers for diagnosing and assessing the severity of lung conditions.

Sputum offers the advantage of being in direct contact with lung tissue, potentially providing a higher “tumor fraction” of exosomes. However, sputum samples are highly variable in volume and composition, often contaminated with saliva, oral microbes, and inflammatory cells, which can complicate exosome isolation (Welsh et al., 2024). To improve reproducibility and minimize contamination, standardized protocols for sputum collection, processing, and isolation are necessary. As with other biofluids, following MISEV guidelines and reporting all relevant metadata on sample collection and processing is essential to ensure consistency and transparency across studies (Welsh et al., 2024). Consequently, although this source is promising, existing data are preliminary, and large, well-controlled validation studies with rigorous methods are needed to confirm utility for early lung cancer diagnosis.

## 5 Exosome as a diagnostic biomarker in early lung cancer diagnosis

**FIGURE 2:**
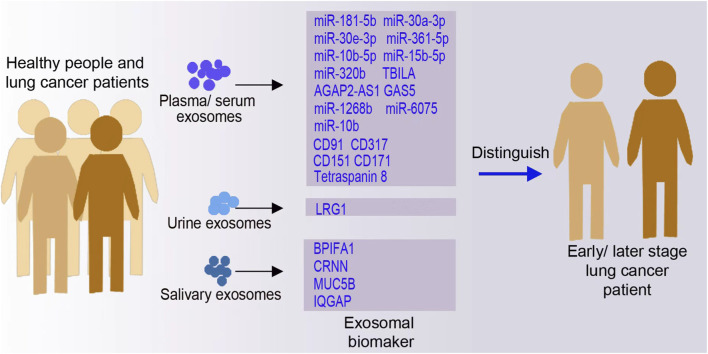
Exosomes for lung cancer diagnosis through exosomal RNAs and proteins. Exosomes, derived from various body fluids such as blood, sputum, and urine, carry important biomarker signatures, including microRNAs and proteins, that can be used to distinguish between different types of lung cancer. Representative biomarkers and concepts illustrated in this figure are supported by studies cited in references (Jakobsen et al., 2015; Vykoukal et al., 2017; Sun et al., 2018; Yuan et al., 2021; Li et al., 2019a; Tao et al., 2020; Sandfeld-Paulsen et al., 2016; Li et al., 2011; Fan et al., 2018). (GAS5: long non-coding RNA growth arrest-specific transcript 5; LRG1: leucine-rich α-2-glycoprotein).

Early diagnosis of lung cancer is crucial, as it is linked to favorable prognostic outcomes. However, the majority of lung cancer cases are diagnosed at advanced stages due to the absence of distinct symptoms in the early stages, resulting in most definitive diagnoses occurring at later stages (Medford et al., 2024; Silva et al., 2024). Research indicates that cancerous lesions smaller than 1 mm may be associated with the presence of circulating tumor cells before they become detectable by imaging, which has a sensitivity threshold of 3–4 mm (Rhim et al., 2012). Various diagnostic methodologies are currently under investigation to enhance the specificity and sensitivity of lung cancer detection, thereby improving both early diagnosis and prognosis. A particularly promising approach involves the utilization of circulating exosomes and their associated cargoes, including miRNAs, proteins, and lipids. In a study focused on the early diagnosis of NSCLC and the differentiation between AdCa and SCC, researchers identified 11 highly expressed and 6 lowly expressed miRNAs in AdCa patients, as well as 13 highly expressed and 8 lowly expressed miRNAs in SCC patients, compared to healthy controls. Subsequent validation of distinct exosomal miRNAs specific to AdCa and SCC revealed that miR-181-5p, miR-30a-3p, miR-30e-3p, and miR-361-5p were specific to AdCa, whereas miR-10b-5p, miR-15b-5p, and miR-320b were specific to SCC (Jin et al., 2017). Figure 2 represents sources of exosomes for lung cancer diagnosis through exosomal biomarker detection.

### 5.1 Exosomal RNAs as biomarkers in early detection

The exosomal lipid bilayer’s ability to protect miRNAs from degradation by cellular RNases in bodily fluids renders exosomal miRNAs an ideal source of non-invasive biomarkers for the early detection and prognosis of cancer (Linares-Rodríguez et al., 2025). The resemblance between circulating exosomal miRNAs and tumor-derived miRNAs enables the use of circulating exosomal miRNAs as a screening tool for lung cancer. A multicenter study including 3,102 participants derived from case–control and cohort datasets, using genome-wide miRNA profiling of blood samples, reported that a 15-miRNA signature distinguished individuals with lung cancer from non-cancer controls in a validation cohort, achieving an accuracy of 91.4% (95% CI, 91.0–91.9), a specificity of 93.5% (95% CI, 93.2–93.8), and a sensitivity of 82.8% (95% CI, 81.5–84.1) (Fehlmann et al., 2020). In the same study, a distinct 14-miRNA panel discriminated lung cancer patients from individuals with non-malignant lung diseases, with an accuracy of 92.5% (95% CI, 92.1–92.9), a specificity of 88.6% (95% CI, 88.1–89.2), and a sensitivity of 96.4% (95% CI, 95.9–96.9) (Fehlmann et al., 2020).

A separate serum-based investigation constructed a diagnostic model based on miR-1268b and miR-6075 using 2,588 miRNA expression profiles, and evaluated this model in a validation cohort of 1,358 lung cancer patients and 1,970 non-cancer participants, reporting both sensitivity and specificity of 99% (Asakura et al., 2020). These findings support the potential diagnostic value of exosomal miRNA signatures. Nevertheless, variations in study design, cohort composition, and validation strategies across studies may influence the reported performance and should be considered when interpreting these results. In particular, highly selected populations and modeling strategies may contribute to optimistic estimates of diagnostic accuracy. Further evaluation in large, prospective screening cohorts will be critical to determine their real-world clinical performance.

Beyond miRNAs, extracellular vesicle-associated miR-10b has been reported to show strong diagnostic performance for lung AdCa, achieving an AUC of 0.998 when compared with established serum tumor markers, including AFP, CEA, CYFRA21-1, NSE, and others (Yuan et al., 2021). In addition, the exosomal long non-coding RNA growth arrest-specific transcript 5 (GAS5) was found to be downregulated in non-small cell lung cancer compared with healthy controls, and the combination of exosomal GAS5 with CEA yielded an AUC of 0.929, with more modest performance for stage I disease (AUC 0.822) (Li et al., 2019a).

Exosomal lncRNAs AGAP2-AS1 and TBILA have also been reported to be overexpressed in non-small cell lung cancer compared with healthy individuals. Receiver operating characteristic analyses indicated moderate discriminatory performance, with reported sensitivity and specificity values ranging from approximately 65%–67% and 73%–81% (Tao et al., 2020), respectively. Although these findings are encouraging, differences in study populations, sample sizes, and analytical approaches across studies highlight the need for further validation. These observations underscore the importance of well-designed prospective screening studies to minimize potential bias and to establish the clinical utility of exosome-based biomarkers for early lung cancer detection.

### 5.2 Exosomal proteins as biomarkers in early detection

Exosomes are known to contain specific surface and intracellular protein molecules that are implicated in the initiation and progression of lung cancer. Consequently, they are being investigated as potential biomarkers for the early diagnosis and prognosis of this disease (Kujtan and Subramanian, 2019). Several exosomal membrane protein markers, including EGFR, CD91, and CD317, have been identified as potential diagnostic biomarkers for lung cancer (Luo et al., 2020). In evaluating the utility of exosomal protein profiling for diagnosing lung cancers across all stages, the researcher isolated plasma exosomes from a cohort of 581 participants, comprising 431 individuals with lung cancer and 150 control subjects. The findings indicated that the markers CD151, CD171, and tetraspanin 8 were the most effective in distinguishing patients with cancer, regardless of histological subtype, from those without cancer. Additionally, a multi-marker model incorporating ten markers achieved an AUC of 0.76 specifically for AdCa and an AUC of 0.74 for all lung cancer histological subtypes, underscoring the potential of exosomal protein profiling in the early diagnosis of lung cancer (Sandfeld-Paulsen et al., 2016). A study examining the correlation between exosomes derived from NSCLC and potential protein markers utilized unique Raman scattering profiles and principal component analysis (PCA) for cancer diagnosis. This research identified distinct peaks that showed a strong correlation with the ratio of cancerous exosomes (R^2^ > 90%), representing a unique Raman band specific to NSCLC exosomes. Further analysis of the Raman bands in conjunction with exosomal protein markers (CD9, EpCAM, CD81, and EGFR) demonstrated that EGFR exhibited a 1.97-fold greater similarity in Raman profiles compared to the other markers (Shin et al., 2018). These findings suggest the potential role of exosomal surface protein markers in cancer diagnosis.

Additionally, a Triple SILAC quantitative proteomic approach was employed to investigate differential protein abundance between exosomes from normal bronchial epithelial cells and those from NSCLC, as a potential screening tool for early lung cancer detection. The data revealed a protein profile associated with NSCLC exosomes, including proteins such as EGFR, GRB2, and SRC, among the 721 exosomal proteins quantified (Clark et al., 2016). The enrichment of these exosomal protein markers in NSCLC positions them as promising candidates for lung cancer detection through a multi-marker protein panel. In a separate study, 25 proteins from salivary exosomes and 40 from microvesicles in lung cancer patients were identified as originating from distal organ cells, with 5 out of the 25 and 9 out of the 40 proteins being associated with lung tissue. Proteins such as BPIFA, MUC5B, CRNN, and IQGAP warrant further investigation for their potential role in lung cancer screening (Sun et al., 2018). Additionally, exosomal leucine-rich α-2-glycoprotein (LRG1) has been reported as a potential biomarker for lung cancer detection, capable of effectively distinguishing early-stage lung cancer from normal controls using urine samples (Di Bella and Taverna, 2024; Li et al., 2011).

### 5.3 Exosomal lipids as biomarkers in early detection

The lipid bilayer of exosomes, estimated to be 5 nm in thickness, comprises approximately 53,400 lipid molecules and constitutes about 37% of the total volume of a 70-nm-sized exosome. This bilayer, containing lipid classes such as cholesterol, phosphatidylcholine, sphingomyelin, phosphatidylserine, and phosphatidylethanolamine, plays a crucial role in protecting the internal components from rapid degradation (Zhou et al., 2023). Notwithstanding, there is a paucity of studies addressing the role of exosomal lipids in early tumor detection. However, recent investigations have highlighted the emerging potential of these lipids in cancer screening and diagnosis. For instance, exosomes isolated from patient blood samples have been subjected to direct analysis via MS fingerprinting without any preparatory treatment, facilitating their application in cancer classification through mathematical analysis and enabling the qualitative and semi-quantitative detection of cancer biomarkers (Zhou et al., 2023; Skotland et al., 2023). The lipid profiles of blood plasma exosomes have been investigated for the early detection of NSCLC utilizing ultra-high-resolution Fourier transform mass spectrometry (UHR-FTMS). The researchers employed multivariate statistical methods to identify the top 16 lipids, which included 9 phosphatidylcholines, 2 sphingomyelins, 2 triacylglycerides, 1 cholesterol ester, 1 lysophosphatidylcholine, 1 lysophosphatidylcholine-plasmalogen, and 7 additional lipid features. The findings indicated that the area under the receiver operating characteristic curve (AUROC) for distinguishing early and late-stage cancer from normal subjects, utilizing the selected lipid features, was 0.85 and 0.88 for the Random Forest (RF) model, and 0.79 and 0.77 for the Least Absolute Shrinkage and Selection Operator (LASSO) model, respectively (Fan et al., 2018). Table 1 provides a summary of studies investigating exosomal components in the context of early lung cancer detection.

**TABLE 1:** Exosomal components in early lung cancer diagnosis and prognosis.

Exosomal component	Source	Isolation method	Sample size	Tumor stage	Diagnostic value	References
Exosomal RNAs						
miR-181-5p, miR-30a-3p, miR-30e-3p, miR-361-5p	Plasma	Differential ultracentrifugation	46 stage I NSCLC patients and 42 healthy individuals	AdCa-specific	Noninvasive biomarkers for early NSCLC diagnosis	Jin et al. (2017)
miR-10b-5p, miR-15b-5p, miR-320b	Plasma	Differential ultracentrifugation	46 stage I NSCLC patients and 42 healthy individuals	SCC-specific	Noninvasive biomarkers for early NSCLC diagnosis	Jin et al. (2017)
LncRNA GAS5	Serum	Polymer-based precipitation​ kit	64 NSCLC patients and 40 healthy individuals	Stage I NSCLC	It could distinguish patients with Stage I NSCLC with an AUC of 0.822, but when combined with carcinoembryonic antigen, the AUC increases to 0.929	Li et al. (2019a)
lncRNA TBILA	Serum	Polymer-based precipitation​ kit	150 NSCLC patients and 150 healthy individuals	AdCa and stage I NSCLC	TBILA exhibited a higher discriminatory capacity for all NSCLC patients, stage I NSCLC patients, and AdCa patients	Tao et al. (2020)
AGAP2-AS1	Serum	Polymer-based precipitation​ kit	150 NSCLC patients and 150 healthy individuals	SCC	AGAP2-AS1 displayed a higher AUC in distinguishing SCC patients from healthy controls	Tao et al. (2020)
miR-10b	Plasma	Polymer-based precipitation​ kit	80 AdCa patients and 69 healthy individuals	AdCa	A diagnostic marker for AdCa with an AUC of 0.998	Yuan et al. (2021)
Exosomal proteins						
CD151, CD171, and tetraspanin 8	Plasma	Extracellular Vesicle Microarray	431 lung cancer patients and 150 controls	All histological subtypes	Differentiate cancer patients from non-cancer patients (CD151: AUC = 0.68; CD171: AUC = 0.60; TSPAN8: AUC = 0.60) AUC = 0.76	Sandfeld-Paulsen et al. (2016)
10 multi-marker model	Plasma	Extracellular Vesicle Microarray	431 lung cancer patients and 150 controls	AdCa All stages	AUC = 0.74	Sandfeld-Paulsen et al. (2016)
30-marker model	Plasma	Extracellular Vesicle Array	109 NSCLC patients and 110 controls	IIIa-IV	Differentiate the two groups with a sensitivity of 0.75, specificity of 0.76, an accuracy of 75.3%, and an AUC of 0.83	Jakobsen et al. (2015)
LRG1	Urine	Differential ultracentrifugation	8 NSCLC patients and 10 controls	NSCLC Early-stage (I&II)	Candidate biomarker for NSCLC differentiation from healthy controls	Li et al. (2011)
SRGN, TPM3, THBS1, and HUWE1	Plasma	Density gradient centrifugation	15 AdCa patients and 13 controls	AdCa	Distinguishes AdCa from normal controls, with an AUC of 0.90 (95% CI = 0.76–1)	Vykoukal et al. (2017)
Exosomal lipids						
16 lipids with top importance +7 features including phosphatidyl choline, sphingomyelins, triacylglycerides, cholesterol ester, lysophosphatidylcholine, and lysophosphatidylcholine-plasmalogen	Plasma	Differential ultracentrifugation	91 NSCLC patients and 39 controls	Early-stage, late-stage, and healthy individuals	The Area Under the Receiver Operating Characteristic curve for early and late-stage cancer versus healthy individuals using the selected lipid features was 0.85 and 0.88 for RF and 0.79 and 0.77 for LASSO, respectively	Fan et al. (2018)

## 6 Established techniques for exosome detection in lung cancer diagnosis

Currently, two primary categories of methodologies are commonly utilized for the analysis of exosome components: (a) protein detection techniques, including Western blotting, enzyme-linked immunosorbent assay (ELISA), and proteomics based on liquid chromatography-tandem mass spectrometry (MS), and (b) DNA and RNA detection techniques, such as next-generation sequencing, polymerase chain reaction (PCR), and Northern blotting (Coumans et al., 2017). These well-established methodologies typically necessitate preparatory extraction and digestion of internal components, as well as specific labeling processes to identify the desired exosomal target molecules. Additionally, flow cytometry is a well-established method for exosomal detection. Unlike DNA and RNA detection techniques, certain protein detection methods have the capability to identify intact exosomes via surface membrane markers.

### 6.1 Mass spectrometry (MS)

MS is an analytical technique that ionizes chemical species and separates them according to their mass-to-charge ratio. Due to its high specificity and sensitivity, MS is capable of identifying and characterizing the molecular composition of exosomes, including proteins, lipids, and metabolites (Zhuang et al., 2024). LC-MS/MS has been utilized to conduct comparative proteomic analyses of exosomes and microvesicles in human saliva for lung cancer (Sun et al., 2018), to profile the proteome of human urinary exosomes (Pan et al., 2024), and to analyze plasma exosomes in cancer (Bhavsar et al., 2024). Ultra-high-resolution Fourier transform mass spectrometry (UHR-FTMS) was employed to examine exosomal lipids for the classification of early and late-stage NSCLC, utilizing multivariate statistical methods such as LASSO. This methodological approach successfully differentiated early-stage lung cancer patients from healthy individuals (Fan et al., 2018). Matrix-assisted laser desorption ionization time-of-flight (MALDI-TOF) mass spectrometry (MS) facilitates the detection of intact exosomes, producing exosomal fingerprints within minutes. This rapid detection method is proposed as a promising tool for cancer biomarker research. MALDI-TOF MS enables the analysis of intact exosomes without necessitating their lysis or labeling, thereby serving as a robust instrument for examining biological samples for diverse clinical applications (Duncan et al., 2016). Furthermore, this technique can be employed for exosome proteome profiling by detecting protein extracts, which holds potential for cancer diagnostic and prognostic biomarker identification (Alberca-Del Arco et al., 2024).

### 6.2 Enzyme-linked immunosorbent assay (ELISA)

ELISA, a widely utilized immunological technique for the quantification of antibodies, antigens, proteins, and glycoproteins in biological samples, has been extensively applied in the study of cancer exosomes. The use of ELISA-based methods for the quantification of proteins specific to cancer-derived exosomes, such as CD44, CD44v6, CD9, EpCAM, and CD81, has been well-documented (Giampieri et al., 2019). Additionally, ELISA is employed to measure cytokines and chemokines expressed by exosomes (Wang et al., 2020). The quantification of low concentrations of specific exosomes in minimal volumes of clinical samples holds potential for noninvasive cancer diagnosis and prognosis. Liu et al. developed an immunosorbent assay for the digital quantification of target exosomes utilizing droplet microfluidics. In this method, exosomes are immobilized on magnetic microbeads via sandwich ELISA complexes, which are tagged with an enzymatic reporter that generates a fluorescent signal. This droplet-based single-exosome-counting ELISA technique enables the absolute quantification of cancer-specific exosomes, achieving unprecedented accuracy with a detection limit of 10 enzyme-labeled exosome complexes per microliter (approximately 10^-17 M) (Pengtao et al., 2024). Ongoing research and advancements in this technique have led to the development of more refined models for specific applications, such as tumor screening and diagnosis. For example, a cell surface ELISA in suspension method was evaluated for exosome detection, demonstrating its suitability for identifying exosome particles within biological samples. This method can be adapted to assess exosome interactions with target cells in both diagnostic and therapeutic contexts (Malaguarnera and Cabrera-Pastor, 2024).

### 6.3 Western blotting

Due to the absence of exosome-specific markers, proteins that are ubiquitously enriched in exosomes from various cellular origins are frequently utilized for exosome detection. Consequently, the markers used for exosome detection may differ depending on the originating cells. Typically, Western blot analysis of exosomes employs markers from each of the categories recommended by the International Society for Extracellular Vesicles (ISEV) for the characterization of extracellular vesicles. These include tetraspanins such as CD9, CD63, and CD81; intracellular proteins associated with multivesicular body (MVB) formation, such as tumor susceptibility gene 101 (TSG101); and chaperone proteins such as heat-shock proteins (HSP) 90 and HSP70. Additionally, some detection kits may include antibodies against the marker Alix, also known as PDCD6IP. As a conventional method for protein detection, Western blotting has been extensively employed in numerous studies to evaluate exosomes expressed in lung tumors through their protein constituents (Liang et al., 2020; Li et al., 2019b; Chen et al., 2024). In various applications, Western blotting has been employed to verify the distinct expression of tumor exosomal surface/membrane proteins, such as glypican 1 (GPC-1), glucose transporter 1 (GLUT-1), and disintegrin and metalloproteinase domain-containing protein 10 (ADAM10), which are considered potential biomarkers for cancer diagnosis and prognosis (Xu F. et al., 2024).

### 6.4 Flow cytometry

Flow cytometry is utilized to measure the light scattering and fluorescence signals of individual exosomes. As reported by Kuiper and colleagues, flow cytometry is the sole technique capable of detecting, sizing, and phenotyping millions of extracellular vesicles within a matter of minutes (Bettin et al., 2023). While single-particle measurement using flow cytometry is advantageous, the diminutive size of exosomes and the low abundance of surface antigens pose significant challenges to conventional flow cytometry methods. This has led to the development of vesicle-specific assays and experimental designs tailored for exosomal studies. For example, the implementation of fluorescence-triggered Vesicle Flow Cytometry (VFC), a comprehensive approach for the quantitative assessment of extracellular vesicle number, size, and surface marker expression, addresses these challenges effectively (Kolenc and Maličev, 2024). Quantification is crucial for comprehending the fundamental biological interactions between exosomes and their parent cells, as well as for interpreting exosome signaling. Furthermore, the quantification of the molecular cargo within exosomes via on-bead flow cytometry is imperative for elucidating their role in information transfer and human disease. Recent research has demonstrated that on-bead flow cytometry, standardized for use with conventional cytometers, is an effective method for the detection and quantification of proteins in exosomes isolated from cell line supernatants or cancer patient plasma (Hong et al., 2024). In this method, exosomes isolated through size-exclusion chromatography are captured using biotinylated antibodies specific to exosomal cargo antigens and immobilized on streptavidin-labeled beads. Detection is subsequently achieved with pre-titrated fluorochrome-labeled antibodies of the desired specificity. Additionally, subpopulations of extracellular vesicles and particles derived from cancer cells have been characterized using nano-flow cytometry (Liu et al., 2022).

## 7 Alternative technologies for exosome detection in lung cancer diagnosis

Exosomal components, including proteins such as EGFR, LRP1, and LG3BP, as well as RNAs present in the serum and urine of lung cancer patients, have been associated with the stage and metastasis of the disease (Yasamineh et al., 2024). Liquid biopsies, which facilitate the capture and detection of tumor-related biomarkers in body fluids, hold significant promise for the early diagnosis of cancer. Tumor-derived exosomes are a prime example of such biomaterials, representing potential biomarkers for various cancers. Due to the labor-intensive, time-consuming, and costly nature of traditional exosomal detection methods, such as ELISA, Western blotting, flow cytometry, and mass spectrometry, recent research has focused on the development of liquid biopsy biosensor-based methods. These innovative technologies aim to address the limitations of conventional approaches and offer point-of-care testing solutions.

### 7.1 Surface-enhanced Raman spectroscopy (SERS)

Among the newly developed technologies for the detection of cancer-derived exosomes, Surface-Enhanced Raman Spectroscopy (SERS) is regarded as one of the most sensitive, reliable, and selective methods for non-destructive molecular analysis. This is achieved through the enhancement of electromagnetic fields and/or the formation of charge-transfer states between the chemisorbed analyte molecule and the SERS-active substrate (Su et al., 2023). In the context of lung cancer, researchers observed a significant variation in the SERS signals of proteins, nucleic acids, and lipids, distinguishing not only between exosomes from normal lung tissue and those from cancerous lung tissue but also between exosomes from fibroblasts and epithelial cells. Further investigation demonstrated that exosomes derived from lung cancer exhibited a pronounced protein SERS signal during time-dependent SERS analysis, whereas exosomes from normal lung tissue predominantly showed SERS signals associated with proteins, lipids, and nucleic acids (Lyu et al., 2024). A label-free and highly sensitive approach for classifying exosomes, which integrates SERS with statistical pattern analysis, effectively differentiated exosomes derived from lung cancer cells from those derived from normal cells, achieving a sensitivity of 95.3% and a specificity of 97.3%. Furthermore, Principal Component Analysis indicated that the observed differences were attributable to 11 distinct points in the SERS signals of exosomes from lung cancer cells (Park et al., 2017). Similarly, a recent investigation demonstrated the precise diagnosis of early-stage lung cancer through the application of deep learning-based SERS on exosomes derived from both normal and lung cancer cell lines. The model exhibited an accuracy of 95% and successfully predicted that 90.7% of plasma exosomes (including those from stage I and II cancer patients) exhibited greater similarity to lung cancer cell exosomes compared to the average healthy controls, with an AUC of 0.912 for the entire cohort and 0.910 for stage I patients (Shin et al., 2020). These findings suggest the potential utility of combining exosome analysis with deep learning as a method for early-stage liquid biopsy in lung cancer diagnostics.

### 7.2 Electric field-induced release and measurement (EFIRM)

EFIRM is a technique designed for the selective extraction of exosomes from biofluids, facilitating the release of their cargo for analysis of internal RNA and protein content. This method has been investigated for its potential in the early diagnosis of lung cancer. EFIRM has demonstrated the capability to show that exosome-like microvesicles transport tumor cell-specific mRNA and protein from blood to saliva in a xenografted mouse model of human lung cancer (Cui et al., 2024). Mutations in the EGFR gene are known to predict sensitivity to EGFR-targeted therapies for NSCLC. Recent advancements indicate that EFIRM can detect EGFR mutations with 100% sensitivity in both plasma and saliva samples from NSCLC patients. Deep sequencing analysis of circulating tumor DNA (ctDNA) enriched for the EGFR L858R mutation revealed a significant presence of EGFR L858R ctDNA as ultrashort ctDNA (usctDNA) with sizes ranging from 40 to 60 base pairs in patient plasma and saliva, with the majority of usctDNAs predominantly localized within the exosomal fraction (Wang F. et al., 2024). Saliva-based EFIRM liquid biopsy has been employed alongside other methodologies to serially assess ctDNA in lung AdCa, serving as a predictor of treatment response and resistance (Kim C. et al., 2021), as well as enabling the specific detection of both exosomal RNA and protein biomarker targets (Tu et al., 2015). The EFIRM method was utilized for the analysis of exosomal profiles in mice injected with human lung cancer H640 cells, a cell line engineered to express the exosome marker human CD63-GFP. The findings indicated elevated expression levels of exosomal biomarkers in both serum and saliva samples of the mice injected with human lung cancer cells, suggesting the potential of salivary exosome biomarkers for the detection of distal diseases (Tu et al., 2015).

### 7.3 Lateral flow immunoassays (LFIA)

Lateral flow immunoassay (LFIA), recognized as one of the most prevalent point-of-care testing techniques, is extensively used as portable strips for biological detection due to its rapidity, cost-effectiveness, and specificity for target biomarkers (Moghadam et al., 2015). This appealing immunochromatographic strip test facilitates easy use and rapid detection of exosomes, even by untrained personnel. Recent studies have incorporated this technique into rapid diagnostic test kits for lung cancer, highlighting its advantages of straightforward operation, high sensitivity, and affordability (Zhou et al., 2020). The techniques, which are predicated on the detection of exosomal miRNA in clinical salivary and urine samples, demonstrated high selectivity and sensitivity, aligning with the standard quantitative real-time polymerase chain reaction (qRT-PCR) method, and exhibited a detection limit of 7.76 × 10^9^ (Zhou et al., 2020). This represents a promising strategy for the screening, monitoring, and differentiation of lung cancer exosomes from normal cell exosomes, thereby serving as a potential diagnostic tool. Purified exosomes from human plasma and urine can be detected within 15 min, with a detection limit of 8.54 × 10^5 exosomes/µL when a combination of anti-CD81 and anti-CD9 is employed as capture antibodies, and anti-CD63 labeled with gold nanoparticles is utilized as the detection antibody (Oliveira-Rodríguez et al., 2016). The challenge of low exosome detection by colorimetric-based lateral-flow assays can be addressed by employing highly bright multi-quantum dots embedded in silica-encapsulated nanoparticles (M-QD-SNs), which enhances the sensitivity of the techniques, achieving an exosome detection limit of 117.94 exosomes/μL. Furthermore, the resultant method for exosome detection is not only highly sensitive and quantitative but also accurate, rapid, and selective (Kim H. M. et al., 2021). In the context of tumor-specific exosome detection, particularly in melanoma, techniques such as LFIA and ELISA have demonstrated efficacy in identifying scarce tumor antigens within exosomes. These methods also successfully detected the human NKG2D-Ligand, MICA, within tetraspanin-containing nanovesicles, as reported in study (López-Cobo et al., 2018), This suggests the potential utility of these techniques for analyzing biological samples to identify tumor-derived exosomes.

### 7.4 Microfluidics

Microfluidics, a multidisciplinary field encompassing engineering, chemistry, physics, biochemistry, biotechnology, and nanotechnology, involves the behavior, manipulation, and precise control of fluids confined to small scales where surface forces predominate over volumetric forces. This field has significant practical applications, particularly in the development of systems designed for processing low volumes of fluids, thereby enabling automation, multiplexing, and high-throughput screening, with notable implications for cancer screening (Garcia-Cordero and Maerkl, 2020). An integrative microfluidic platform has been developed for the isolation and ultrasensitive detection of lung cancer-specific exosomes from patient urine. This device comprises a section modified with a capture antibody and a second antibody-conjugated gold (Au) nanorod probe, which is utilized to identify and quantify lung cancer-specific exosomes via dark field microscopy. The resulting AuNC-Exosome-AuR complex induces a notable scattering wavelength shift and enhanced scattering intensity, enabling the ultrasensitive detection of exosomes with a limit of detection (LOD) below 1,000 particles/mL (Di Bella and Taverna, 2024). Validation using 500 μL urine samples from lung cancer patients demonstrated the device’s efficacy in distinguishing early-stage lung cancer patients from healthy controls. Similarly, a magnetic-based microfluidic platform, known as the ExoPCD-chip, has been reported for the on-chip isolation and detection of tumor-derived exosomes. This device efficiently captures exosomes, achieving highly sensitive detection of CD63-positive exosomes at concentrations as low as 4.39 × 10^3 particles/mL (Xu et al., 2018). Utilizing human serum from patients with liver cancer, the ExoPCD-chip demonstrated a robust ability to distinguish between cancer patients and healthy controls. Comparable studies have documented the isolation and profiling of circulating tumor-associated exosomes through the use of an extracellular vesicular lipid-protein binding affinity-based microfluidic device (Kang et al., 2019) and a detachable microfluidic device equipped with an electrochemical aptasensor (DeMEA) for the sequential analysis of cancerous exosomes (Feng et al., 2023).

### 7.5 Other techniques

Numerous alternative methods for exosome detection have been documented and are continually evolving. A recent investigation introduced an electrochemical micro-aptasensor, designed as a highly sensitive approach for exosome detection by integrating a micropatterned electrochemical aptasensor with a hybridization chain reaction (HCR) for signal amplification. In this methodology, exosomes are concentrated on electrodes functionalized with CD63 aptamers and subsequently identified by HCR products linked to avidin-horseradish peroxidase (HRP) via EpCAM aptamers as intermediaries. Signal generation is achieved through the enzymatic reaction between the HRP enzyme and 3,3′,5,5′-tetramethylbenzidine (TMB)/H2O2, which is directly proportional to the quantity of HRP bound to the HCR products, thereby reflecting the concentration of target exosomes (Zhang et al., 2021). This technique exhibits a detection limit of 5 × 10^2^ exosomes/mL and effectively identifies lung cancer exosomes in serum samples from patients with both early-stage and late-stage lung cancer. Alternative ultrasensitive electrochemical aptasensors for tumor exosome detection have been developed utilizing click chemistry, offering a detection range from 1.12 × 10^2^ to 1.12 × 10^8^ particles/μL and a detection limit of 96 particles/μL (An et al., 2019). Additionally, a sensitive and selective colorimetric aptasensor has been engineered for the detection of cancer-derived exosomes, employing HRP-accelerated dopamine polymerization and *in situ* polydopamine deposition, achieving a detection limit of 7.7 × 10^3^ particles/mL, which represents an improvement of 3-5 orders of magnitude over conventional Dot-blot methods (Küçük et al., 2024). Furthermore, the integration of micronuclear magnetic resonance and microfluidics constitutes another sensitive platform for the detection of extracellular vesicles, including exosomes. In this approach, exosomes are preconcentrated, purified, and labeled with magnetic nanoparticles (MNPs), which are then directed into the microfluidics system, where the labeled exosomes are subsequently trapped on a membrane filter (Mohammadi et al., 2021). Table 2 presents some of the alternative techniques and their diagnostic features in lung cancer.

**TABLE 2:** Alternative exosome detection techniques in lung cancer diagnosis.

Diagnostic method/model	Marker(s)	Distinguishing feature	Specificity/sensitivity	References
SERS + PCA	-	Distinguishes lung cancer cell-derived exosomes from normal cell-derived exosomes	Sensitivity of 95.3% Specificity of 97.3%	Park et al. (2017)
EFIRM	-	Detects the abundant existence of EGFR ctDNA in the plasma and saliva of NSCLC patients	Sensitivity of 100%	Wang X. et al. (2024)
LFIA	-	Portable point-of-care testing to diagnose lung cancer via the detection of exosomal miRNA in urine and saliva	Consistent with qRT-PCR results, the elective and sensitive	Zhou et al. (2020)
Microfluidic	LRG1, using capture antibody anti-CD63 and anti-LRG1-AuR	Differentiates early-stage lung cancer patients from healthy individuals using urine	Ultrasensitive detection of exosomes with a LOD below 1,000 exosomes ml^-1^	Di Bella and Taverna (2024)
SERS	Different profiles of proteins, RNAs, and lipids	Differentiates between not only normal lung and lung cancer exosomes but also between fibroblast and epithelium exosomes	distinguishes normal lung and lung cancer exosomes at low concentrations	Lyu et al. (2024)
An electrochemical aptasensor and a hybridization chain reaction (HCR) signal amplification	Detects exosomal CD63	Successfully detects lung cancer exosomes in serum samples of early-stage and late-stage lung cancer patients	Wide range of exosome detection (2.5 × 10^3^ to 1 × 10^7^ exosomes/mL), with a detection limit of 5 × 10^2^ exosomes/mL	Zhang et al. (2021)
Electrochemical aptasensor based on click chemistry and the DNA hybridization chain reaction (HCR)	Detects exosomal CD63	Sensitive and accurate quantification of exosomes in human serum	Exosome detection range of 1.12 × 10^2^ to 1.12 × 10^8^ particles/μL with a limit of detection of 96 particles/μL	An et al. (2019)

## 8 Commercial products for lung cancer exosome detection

Recent advancements in exosome research have led to the development of numerous commercial products and kits that employ a variety of techniques for the reliable isolation and detection of exosomes. Many of these exosome-based products and kits function as highly sensitive and specific tools for cancer diagnosis. These products encompass a range of applications, including exosome isolation, detection, purification, quantification, and profiling from diverse sources. For instance, Thermo Fisher Scientific offers comprehensive exosome isolation products for sources such as plasma, urine, serum, cerebrospinal fluid (CSF), ascitic fluid, amniotic fluid, milk, saliva, and cell culture media. Companies involved in this domain include Exosome Diagnostics Inc., Novus Biologicals, IMEX (Integrated Magnetic-Electrochemical Exosomes), Izon Science Inc., GenExosome Technologies Inc., RoosterBio Inc., HansaBioMed (HBM), Beckman Coulter, System Biosciences (SBI), Bioo Scientific Corporation, Miltenyi Biotec, Excipio Technologies, Norgen Biotek Corporation, and Wako Pure Chemical Industries, Ltd. These companies develop specialized kits that exhibit distinct technical performance characteristics and present various practical challenges. Consequently, selecting the most suitable exosome isolation and detection method for quantification and analysis necessitates a careful evaluation of the advantages and disadvantages associated with each technique. It is crucial to thoroughly examine the background characteristics of the targeted exosomes, such as the type and level of antigen expression on their surface.

In the context of lung cancer diagnostics, specific products such as ExoDx Lung (ALK), ExoDx Lung (T790M), and ExoDx Lung (EGFR) by Exosome Diagnostics Inc. facilitate sensitive, accurate, and real-time mutation detection through exosomes. ExoDx Lung (ALK) accurately identifies the EML4-ALK mutation in cancer cells by analyzing exosomal RNAs in the blood, while ExoDx Lung (T790M) detects the EGFR T790M mutation, and ExoDx Lung (EGFR) identifies both EGFR activation and T790M resistance mutations. In relation to the precision of lung cancer diagnosis, a study demonstrated that ExoDx (EGFR) exhibits a sensitivity of 90% for L858R, 83% for T790M, and 73% for exon 19 indels, with corresponding specificities of 100%, 100%, and 96%, respectively (Mohammadi et al., 2021). Alternative products, such as exoEasy and exoRNeasy developed by Qiagen and Exosome Diagnostics Inc., employ membrane affinity spin columns to effectively isolate exosomes and other extracellular vesicles from various biofluids. These methods accommodate both small sample volumes (up to 1 mL of serum/plasma or 4 mL of urine) and larger volumes (up to 4 mL of serum/plasma, 16 mL of urine, or 32 mL of cell culture supernatant). Additionally, Novus Biologicals offers immunoplates and immunobeads designed to capture exosomes via plates pre-coated with proprietary pan-exosome marker antibodies, utilizing a double sandwich ELISA for quantification. These chips require only 100 μL of the sample. IMEX kits utilize electrochemical detection of surface markers, such as CD63, whereby exosomes in patient samples are immunomagnetically labeled, captured, and assessed using electrochemical sensors (Mohammadi et al., 2021).

## 9 Challenges, future perspectives, and conclusion

Current diagnostic approaches for lung cancer are challenged by low sensitivity, limited tumor information due to heterogeneity and inadequate sampling, high false-positive rates, invasive or uncomfortable procedures, and substantial costs. As research into liquid biopsies that analyze circulating biomarkers in body fluids gains prominence, exosomes have emerged as a promising solution to these issues. These nano-sized extracellular vesicles are capable of carrying and transferring specific biomaterials, including RNAs and proteins. In recent years, the application of standardized analytical methods and high-throughput omics technologies in exosome biomarker research has led to the identification of numerous potential exosome-based biomarkers for various diseases, including lung cancer. Significant advancements have been made in the development of liquid biopsy biosensors, enabling rapid, highly sensitive, and high-throughput analysis of exosomes. The successful detection of exosomal components in biospecimens such as serum and plasma, serving as biomarkers in cancer patients, underscores the potential application of biosensors in clinical settings. To advance the development of exosome-based biosensors into precise and sensitive routine clinical diagnostic assays, sustained efforts are imperative.

Despite advancements in exosome research, their application in the early diagnosis of lung cancer remains distant from achieving the ideal early diagnostic tool. As highlighted by the Urine Task Force of the Rigor and Standardization Subcommittee of the International Society for Extracellular Vesicles, there is a pressing need for further optimization and standardization of methodological aspects related to extracellular vesicle separation and analysis, including result normalization, to promote scientific progress in urine extracellular vesicle research and facilitate its successful clinical translation (Erdbrügger et al., 2021). This necessity extends to all sources of extracellular vesicles, aiming to enhance the rigor, reproducibility, and interoperability of exosomal research. Such improvements would create a conducive environment for discovering highly specific and sensitive screening and diagnostic methods for lung cancer, utilizing exosomes as biomarkers.

The inherent heterogeneity of exosomes poses a significant challenge in establishing their analytical and clinical validity. The co-isolation of vesicles originating from non-tumor cells within the tumor microenvironment, such as fibroblasts and immune cells, can produce background signals that obscure tumor-specific signatures. Standardization is a critical obstacle; variations in isolation techniques—such as ultracentrifugation, precipitation, and immunoaffinity capture—can selectively enrich different exosome subpopulations, resulting in inconsistent findings across studies. Therefore, biomarker validation necessitates not only confirming the presence of a biomarker but also identifying the specific exosomal subpopulation in which it resides and ensuring robust, reproducible measurement across platforms. Addressing these challenges is essential to fully exploit the potential of exosome heterogeneity, ultimately facilitating their clinical translation as liquid biopsy tools for the personalized management of lung cancer patients.

Given the complexity inherent in biological samples and the tumor microenvironment, future research should focus on developing biosensor techniques that can accurately identify exosomes amidst all sample components. These methods should also be capable of distinguishing exosomes from other extracellular vesicles, such as apoptotic bodies and microvesicles, which complicate exosome detection due to shared surface markers and overlapping size ranges. It is imperative to explore the integration of exosomal components into a unified diagnostic array. Specifically, the combination of exosomal surface protein biomarkers with intra-vesicular proteins and RNAs could yield a more comprehensive profile of early-stage lung tumors, thereby enhancing the diagnostic utility of exosomes. Furthermore, the promising exosomal biomarkers identified must be validated in a large cohort of lung cancer patients to substantiate their clinical benefits. In summary, exosome-based liquid biopsies represent promising methodologies that could complement early lung cancer screening and diagnosis, as well as prognosis, personalized therapy, and monitoring of treatment responses in lung cancer. Despite being in the nascent stages of exploration and facing numerous challenges that must be addressed for clinical implementation, the progress achieved thus far suggests a potentially substantial impact on lung cancer care.

Limitations of this review include the potential for the selection and synthesis of studies being influenced by subjective judgment, and may not fully capture the breadth of the existing literature. Publication bias is also a concern, as positive or promising findings on exosome-based biomarkers in lung cancer are more likely to be published than negative or null results, potentially overstating the apparent diagnostic value.

## References

[B1] Adams S. J. Stone E. Baldwin D. R. Vliegenthart R. Lee P. Fintelmann F. J. (2023). Lung cancer screening. Lancet. 401, 390–408. 10.1016/S0140-6736(22)01694-4 36563698

[B2] Alberca-Del Arco F. Prieto-Cuadra D. Santos-Perez de la Blanca R. Sáez-Barranquero F. Matas-Rico E. Herrera-Imbroda B. (2024). New perspectives on the role of liquid biopsy in bladder cancer: applicability to precision medicine. Cancers (Basel) 16, 803. 10.3390/cancers16040803 38398192 PMC10886494

[B3] An Y. Jin T. Zhu Y. Zhang F. He P. (2019). An ultrasensitive electrochemical aptasensor for the determination of tumor exosomes based on click chemistry. Biosens. Bioelectron. 142, 111503. 10.1016/j.bios.2019.111503 31376716

[B4] Asakura K. Kadota T. Matsuzaki J. Yoshida Y. Yamamoto Y. Nakagawa K. (2020). A miRNA-based diagnostic model predicts resectable lung cancer in humans with high accuracy. Commun. Biol. 3, 134. 10.1038/s42003-020-0863-y 32193503 PMC7081195

[B5] Ayala-Mar S. Donoso-Quezada J. González-Valdez J. (2021). Clinical implications of exosomal PD-L1 in cancer immunotherapy. J. Immunol. Res. 2021, 8839978. 10.1155/2021/8839978 33628854 PMC7886511

[B6] Bettin B. A. Varga Z. Nieuwland R. van der Pol E. (2023). Standardization of extracellular vesicle concentration measurements by flow cytometry: the past, present, and future. J. Thromb. Haemost. 21, 2032–2044. 10.1016/j.jtha.2023.04.042 37201724

[B7] Bhavsar D. Raguraman R. Kim D. Ren X. Munshi A. Moore K. (2024). Exosomes in diagnostic and therapeutic applications of ovarian cancer. J. Ovarian Res. 17, 113. 10.1186/s13048-024-01417-0 38796525 PMC11127348

[B8] Bitter E. E. Skidmore J. Allen C. I. Erickson R. I. Morris R. M. Mortimer T. (2023). TK1 expression influences pathogenicity by cell cycle progression, cellular migration, and cellular survival in HCC 1806 breast cancer cells. PLoS One 18, e0293128. 10.1371/journal.pone.0293128 38033034 PMC10688958

[B9] Bojović K. Ignjatović Ð. I. Soković Bajić S. Vojnović Milutinović D. Tomić M. Golić N. (2020). Gut microbiota dysbiosis associated with altered production of short chain fatty acids in children with neurodevelopmental disorders. Front. Cell Infect. Microbiol. 10, 223. 10.3389/fcimb.2020.00223 32509596 PMC7248180

[B10] Bray F. Laversanne M. Sung H. Ferlay J. Siegel R. L. Soerjomataram I. (2024). Global cancer statistics 2022: GLOBOCAN estimates of incidence and mortality worldwide for 36 cancers in 185 countries. CA Cancer J. Clin. 74, 229–263. 10.3322/caac.21834 38572751

[B11] Castillo-Peña A. Molina-Pinelo S. (2023). Landscape of tumor and immune system cells-derived exosomes in lung cancer: mediators of antitumor immunity regulation. Front. Immunol. 14, 1279495. 10.3389/fimmu.2023.1279495 37915578 PMC10616833

[B12] Chatterjee S. Chatterjee A. Jana S. Dey S. Roy H. Das M. K. (2021). Transforming growth factor beta orchestrates PD-L1 enrichment in tumor-derived exosomes and mediates CD8 T-cell dysfunction regulating early phosphorylation of TCR signalome in breast cancer. Carcinogenesis 42, 38–47. 10.1093/carcin/bgaa092 32832992

[B13] Chen C. Y. Yang S. H. Chang P. Y. Chen S. F. Nieh S. Huang W. Y. (2024). Cancer-associated-fibroblast-mediated paracrine and autocrine SDF-1/CXCR4 signaling promotes stemness and aggressiveness of colorectal cancers. Cells 13, 1334. 10.3390/cells13161334 39195225 PMC11352219

[B14] Clark D. J. Fondrie W. E. Yang A. Mao L. (2016). Triple SILAC quantitative proteomic analysis reveals differential abundance of cell signaling proteins between normal and lung cancer-derived exosomes. J. Proteomics 133, 161–169. 10.1016/j.jprot.2015.12.023 26739763

[B15] Coumans F. A. W. Brisson A. R. Buzas E. I. Dignat-George F. Drees E. E. E. El-Andaloussi S. (2017). Methodological guidelines to study extracellular vesicles. Circ. Res. 120, 1632–1648. 10.1161/CIRCRESAHA.117.309417 28495994

[B16] Cui L. Zheng J. Lu Y. Lin P. Lin Y. Zheng Y. (2024). New frontiers in salivary extracellular vesicles: transforming diagnostics, monitoring, and therapeutics in oral and systemic diseases. J. Nanobiotechnology 22, 171. 10.1186/s12951-024-02443-2 38610017 PMC11015696

[B17] Di Bella M. A. Taverna S. (2024). Extracellular vesicles: diagnostic and therapeutic applications in cancer. Biol. (Basel). 13, 716. 10.3390/biology13090716 39336143 PMC11446462

[B18] Duncan M. W. Nedelkov D. Walsh R. Hattan S. J. (2016). Applications of MALDI mass spectrometry in clinical chemistry. Clin. Chem. 62, 134–143. 10.1373/clinchem.2015.239491 26585930

[B19] Erdbrügger U. Blijdorp C. J. Bijnsdorp I. V. Borràs F. E. Burger D. Bussolati B. (2021). Urinary extracellular vesicles: a position paper by the urine task force of the international society for extracellular vesicles. J. Extracell. Vesicles 10, e12093. 10.1002/jev2.12093 34035881 PMC8138533

[B20] Fan T. W. M. Zhang X. Wang C. Yang Y. Kang W. Y. Arnold S. (2018). Exosomal lipids for classifying early and late stage non-small cell lung cancer. Anal. Chim. Acta 1037, 256–264. 10.1016/j.aca.2018.02.051 30292300 PMC6582997

[B21] Fan J. Xu G. Chang Z. Zhu L. Yao J. (2020). miR-210 transferred by lung cancer cell-derived exosomes may act as proangiogenic factor in cancer-associated fibroblasts by modulating JAK2/STAT3 pathway. Clin. Sci. (Lond). 134, 807–825. 10.1042/CS20200039 32219336

[B22] Fehlmann T. Kahraman M. Ludwig N. Backes C. Galata V. Keller V. (2020). Evaluating the use of circulating MicroRNA profiles for lung cancer detection in symptomatic patients. JAMA Oncol. 6, 714–723. 10.1001/jamaoncol.2020.0001 32134442 PMC7059111

[B23] Feng W. Xu P. Wang M. Wang G. Li G. Jing A. (2023). Electrochemical micro-immunosensor of cubic AuPt dendritic Nanocrystals/Ti(3)C(2)-MXenes for exosomes detection. Micromachines (Basel). 14, 138. 10.3390/mi14010138 36677199 PMC9864933

[B24] Fox A. H. Nishino M. Osarogiagbon R. U. Rivera M. P. Rosenthal L. S. Smith R. A. (2023). Acquiring tissue for advanced lung cancer diagnosis and comprehensive biomarker testing: a national lung cancer roundtable best-practice guide. CA Cancer J. Clin. 73, 358–375. 10.3322/caac.21774 36859638 PMC12453630

[B25] Garcia-Cordero J. L. Maerkl S. J. (2020). Microfluidic systems for cancer diagnostics. Curr. Opin. Biotechnol. 65, 37–44. 10.1016/j.copbio.2019.11.022 31891869

[B26] Giampieri R. Piva F. Occhipinti G. Bittoni A. Righetti A. Pagliaretta S. (2019). Clinical impact of different exosomes' protein expression in pancreatic ductal carcinoma patients treated with standard first line palliative chemotherapy. PLoS One 14, e0215990. 10.1371/journal.pone.0215990 31048929 PMC6497273

[B27] Grannis F. W. Jr (2022). Limitations of molecular testing in combination with computerized tomographic for lung cancer screening. Nat. Commun. 13, 3890. 10.1038/s41467-022-31419-9 35803922 PMC9270395

[B28] Guiot J. Cambier M. Boeckx A. Henket M. Nivelles O. Gester F. (2020). Macrophage-derived exosomes attenuate fibrosis in airway epithelial cells through delivery of antifibrotic miR-142-3p. Thorax 75, 870–881. 10.1136/thoraxjnl-2019-214077 32759383 PMC7509395

[B29] Hanjani N. A. Esmaelizad N. Zanganeh S. Gharavi A. T. Heidarizadeh P. Radfar M. (2022). Emerging role of exosomes as biomarkers in cancer treatment and diagnosis. Crit. Rev. Oncol. Hematol. 169, 103565. 10.1016/j.critrevonc.2021.103565 34871719

[B30] Harada T. Yamamoto H. Kishida S. Kishida M. Awada C. Takao T. (2017). Wnt5b-associated exosomes promote cancer cell migration and proliferation. Cancer Sci. 108, 42–52. 10.1111/cas.13109 27762090 PMC5276837

[B31] He B. X. Fang S. B. Xie Y. C. Lou D. X. Wu Z. C. Li C. G. (2024). Small extracellular vesicles derived from human mesenchymal stem cells prevent Th17-dominant neutrophilic airway inflammation *via* immunoregulation on Th17 cells. Int. Immunopharmacol. 133, 112126. 10.1016/j.intimp.2024.112126 38669946

[B32] Hendrick R. E. Smith R. A. (2024). Benefit-to-radiation-risk of low-dose computed tomography lung cancer screening. Cancer 130, 216–223. 10.1002/cncr.34855 37909872

[B33] Hisakane K. Seike M. Sugano T. Yoshikawa A. Matsuda K. Takano N. (2021). Exosome-derived miR-210 involved in resistance to osimertinib and epithelial-mesenchymal transition in EGFR mutant non-small cell lung cancer cells. Thorac. Cancer 12, 1690–1698. 10.1111/1759-7714.13943 33939301 PMC8169289

[B34] Hong W. Xue M. Jiang J. Zhang Y. Gao X. (2020). Circular RNA circ-CPA4/let-7 miRNA/PD-L1 axis regulates cell growth, stemness, drug resistance and immune evasion in non-small cell lung cancer (NSCLC). J. Exp. Clin. Cancer Res. 39, 149. 10.1186/s13046-020-01648-1 32746878 PMC7397626

[B35] Hong C. S. Diergaarde B. Whiteside T. L. (2024). Small extracellular vesicles in plasma carry luminal cytokines that remain undetectable by antibody-based assays in cancer patients and healthy donors. BJC Rep. 2, 16. 10.1038/s44276-024-00037-x 38938748 PMC11210721

[B36] Hoshino A. Costa-Silva B. Shen T. L. Rodrigues G. Hashimoto A. Tesic Mark M. (2015). Tumour exosome integrins determine organotropic metastasis. Nature 527, 329–335. 10.1038/nature15756 26524530 PMC4788391

[B37] Huang H. Yang Y. Zhu Y. Chen H. Yang Y. Zhang L. (2022). Blood protein biomarkers in lung cancer. Cancer Lett. 551, 215886. 10.1016/j.canlet.2022.215886 35995139

[B38] Huang G. Zheng W. Zhou Y. Wan M. Hu T. (2024). Recent advances to address challenges in extracellular vesicle-based applications for lung cancer. Acta Pharm. Sin. B 14, 3855–3875. 10.1016/j.apsb.2024.06.010 39309489 PMC11413688

[B39] Jakobsen K. R. Paulsen B. S. Bæk R. Varming K. Sorensen B. S. Jørgensen M. M. (2015). Exosomal proteins as potential diagnostic markers in advanced non-small cell lung carcinoma. J. Extracell. Vesicles 4, 26659. 10.3402/jev.v4.26659 25735706 PMC4348413

[B40] Jin X. Chen Y. Chen H. Fei S. Chen D. Cai X. (2017). Evaluation of tumor-derived exosomal miRNA as potential diagnostic biomarkers for early-stage non-small cell lung cancer using next-generation sequencing. Clin. Cancer Res. 23, 5311–5319. 10.1158/1078-0432.CCR-17-0577 28606918

[B41] Kang Y. T. Purcell E. Palacios-Rolston C. Lo T. W. Ramnath N. Jolly S. (2019). Isolation and profiling of circulating tumor-associated exosomes using extracellular vesicular lipid-protein binding affinity based microfluidic device. Small 15, e1903600. 10.1002/smll.201903600 31588683 PMC6951813

[B42] Kerpel-Fronius A. (2022). Low-dose lung cancer screening programs - european outlook. Magy. Onkol. 66, 202–206. 36200500

[B43] Kewitz-Hempel S. Windisch N. Hause G. Müller L. Sunderkötter C. Gerloff D. (2024). Extracellular vesicles derived from melanoma cells induce carcinoma-associated fibroblasts *via* miR-92b-3p mediated downregulation of PTEN. J. Extracell. Vesicles 13, e12509. 10.1002/jev2.12509 39315679 PMC11420832

[B44] Khan I. A. Saraya A. (2023). Circulating MicroRNAs as noninvasive diagnostic and prognostic biomarkers in pancreatic cancer: a review. J. Gastrointest. Cancer 54, 720–730. 10.1007/s12029-022-00877-1 36322366

[B45] Kim J. Kim T. Y. Lee M. S. Mun J. Y. Ihm C. Kim S. A. (2016). Exosome cargo reflects TGF-β1-mediated epithelial-to-mesenchymal transition (EMT) status in A549 human lung adenocarcinoma cells. Biochem. Biophys. Res. Commun. 478, 643–648. 10.1016/j.bbrc.2016.07.124 27492069

[B46] Kim C. Xi L. Cultraro C. M. Wei F. Jones G. Cheng J. (2021). Longitudinal circulating tumor DNA analysis in blood and saliva for prediction of response to osimertinib and disease progression in EGFR-mutant lung adenocarcinoma. Cancers (Basel) 13, 3342. 10.3390/cancers13133342 34283064 PMC8268167

[B47] Kim H. M. Oh C. An J. Baek S. Bock S. Kim J. (2021). Multi-quantum dots-embedded silica-encapsulated nanoparticle-based lateral flow assay for highly sensitive exosome detection. Nanomater. (Basel) 11, 768. 10.3390/nano11030768 33803623 PMC8002883

[B48] Kolenc A. Maličev E. (2024). Current methods for analysing mesenchymal stem cell-derived extracellular vesicles. Int. J. Mol. Sci. 25, 3439. 10.3390/ijms25063439 38542411 PMC10970230

[B49] Küçük B. N. Yilmaz E. G. Aslan Y. Erdem Ö. Inci F. (2024). Shedding light on cellular secrets: a review of advanced optical biosensing techniques for detecting extracellular vesicles with a special focus on cancer diagnosis. ACS Appl. Bio Mater 7, 5841–5860. 10.1021/acsabm.4c00782 39175406 PMC11409220

[B50] Kujtan L. Subramanian J. (2019). Epidermal growth factor receptor tyrosine kinase inhibitors for the treatment of non-small cell lung cancer. Expert Rev. Anticancer Ther. 19, 547–559. 10.1080/14737140.2019.1596030 30913927

[B51] Li Y. Zhang Y. Qiu F. Qiu Z. (2011). Proteomic identification of exosomal LRG1: a potential urinary biomarker for detecting NSCLC. Electrophoresis 32, 1976–1983. 10.1002/elps.201000598 21557262

[B52] Li C. Lv Y. Shao C. Chen C. Zhang T. Wei Y. (2019a). Tumor-derived exosomal lncRNA GAS5 as a biomarker for early-stage non-small-cell lung cancer diagnosis. J. Cell Physiol. 234, 20721–20727. 10.1002/jcp.28678 31032916

[B53] Li C. Li C. Zhi C. Liang W. Wang X. Chen X. (2019b). Clinical significance of PD-L1 expression in serum-derived exosomes in NSCLC patients. J. Transl. Med. 17, 355. 10.1186/s12967-019-2101-2 31665020 PMC6820965

[B54] Li K. Xue W. Lu Z. Wang S. Zheng J. Lu K. (2024). Tumor-derived exosomal ADAM17 promotes pre-metastatic niche formation by enhancing vascular permeability in colorectal cancer. J. Exp. Clin. Cancer Res. 43, 59. 10.1186/s13046-024-02991-3 38413999 PMC10898123

[B55] Liang M. Chen X. Wang L. Qin L. Wang H. Sun Z. (2020). Cancer-derived exosomal TRIM59 regulates macrophage NLRP3 inflammasome activation to promote lung cancer progression. J. Exp. Clin. Cancer Res. 39, 176. 10.1186/s13046-020-01688-7 32867817 PMC7457778

[B56] Linares-Rodríguez M. Blancas I. Rodríguez-Serrano F. (2025). The predictive value of blood-derived exosomal miRNAs as biomarkers in breast cancer: a systematic review. Clin. Breast Cancer 25, e48–e55.e15. 10.1016/j.clbc.2024.06.016 39054208

[B57] Liu H. Tian Y. Xue C. Niu Q. Chen C. Yan X. (2022). Analysis of extracellular vesicle DNA at the single-vesicle level by nano-flow cytometry. J. Extracell. Vesicles 11, e12206. 10.1002/jev2.12206 35373518 PMC8977970

[B58] Liu J. Zhang F. Wang J. Wang Y. (2023). MicroRNA-mediated regulation in lung adenocarcinoma: signaling pathways and potential therapeutic implications. Oncol. Rep. 50, 211. 10.3892/or.2023.8648 37859595 PMC10603552

[B59] Liu X. Wu F. Pan W. Liu G. Zhang H. Yan D. (2024). Tumor-associated exosomes in cancer progression and therapeutic targets. MedComm (2020) 5, e709. 10.1002/mco2.709 39247621 PMC11380050

[B60] Liu F. Zhang X. Lu M. Liu C. Zhang X. Chu Q. (2024). The association of genomic alterations with PD-L1 expression in Chinese patients with EGFR/ALK wild-type lung adenocarcinoma and potential predictive value of hippo pathway mutations to immunotherapy. Cancer Med. 13, e7038. 10.1002/cam4.7038 38396367 PMC10891359

[B61] López-Cobo S. Campos-Silva C. Moyano A. Oliveira-Rodríguez M. Paschen A. Yáñez-Mó M. (2018). Immunoassays for scarce tumour-antigens in exosomes: detection of the human NKG2D-Ligand, MICA, in tetraspanin-containing nanovesicles from melanoma. J. Nanobiotechnology 16, 47. 10.1186/s12951-018-0372-z 29720199 PMC5932892

[B62] Lu C. Shan Z. Hong J. Yang L. (2017). MicroRNA-92a promotes epithelial-mesenchymal transition through activation of PTEN/PI3K/AKT signaling pathway in non-small cell lung cancer metastasis. Int. J. Oncol. 51, 235–244. 10.3892/ijo.2017.3999 28534966

[B63] Luo D. Li C. Wu L. Chen Q. (2020). Advances of exosomes extraction and its mechanism in early diagnosis of lung cancer. Zhongguo Fei Ai Za Zhi 23, 999–1006. 10.3779/j.issn.1009-3419.2020.101.24 32752584 PMC7679221

[B64] Lv J. Xiong X. (2024). Extracellular vesicle microRNA: a promising biomarker and therapeutic target for respiratory diseases. Int. J. Mol. Sci. 25, 9147. 10.3390/ijms25179147 39273095 PMC11395461

[B65] Lyu N. Hassanzadeh-Barforoushi A. Rey Gomez L. M. Zhang W. Wang Y. (2024). SERS biosensors for liquid biopsy towards cancer diagnosis by detection of various circulating biomarkers: current progress and perspectives. Nano Converg. 11, 22. 10.1186/s40580-024-00428-3 38811455 PMC11136937

[B66] Malaguarnera M. Cabrera-Pastor A. (2024). Emerging role of extracellular vesicles as biomarkers in neurodegenerative diseases and their clinical and therapeutic potential in central nervous system pathologies. Int. J. Mol. Sci. 25, 10068. 10.3390/ijms251810068 39337560 PMC11432603

[B67] Martínez-Espinosa I. Serrato J. A. Cabello-Gutiérrez C. Carlos-Reyes Á. Ortiz-Quintero B. (2024). Exosome-derived miRNAs in liquid biopsy for lung cancer. Life 14, 1608. 10.3390/life14121608 39768316 PMC11678223

[B68] Medford A. J. Carmeli A. B. Ritchie A. Wagle N. Garraway L. Lander E. S. (2024). A standing platform for cancer drug development using ctDNA-based evidence of recurrence. Nat. Rev. Cancer 24, 810–821. 10.1038/s41568-024-00742-2 39349822

[B69] Moghadam B. Y. Connelly K. T. Posner J. D. (2015). Two orders of magnitude improvement in detection limit of lateral flow assays using isotachophoresis. Anal. Chem. 87, 1009–1017. 10.1021/ac504552r 25495988

[B70] Mohammadi M. Zargartalebi H. Salahandish R. Aburashed R. Wey Yong K. Sanati-Nezhad A. (2021). Emerging technologies and commercial products in exosome-based cancer diagnosis and prognosis. Biosens. Bioelectron. 183, 113176. 10.1016/j.bios.2021.113176 33845291

[B71] Mortezaee K. Majidpoor J. Kharazinejad E. (2023). The impact of hypoxia on tumor-mediated bypassing anti-PD-(L)1 therapy. Biomed. Pharmacother. 162, 114646. 10.1016/j.biopha.2023.114646 37011483

[B72] Niu Y. Su M. Wu Y. Fu L. Kang K. Li Q. (2019). Circulating plasma miRNAs as potential biomarkers of non-small cell lung cancer obtained by high-throughput real-time PCR profiling. Cancer Epidemiol. Biomarkers Prev. 28, 327–336. 10.1158/1055-9965.EPI-18-0723 30377207

[B73] Njock M. S. Guiot J. Henket M. A. Nivelles O. Thiry M. Dequiedt F. (2019). Sputum exosomes: promising biomarkers for idiopathic pulmonary fibrosis. Thorax 74, 309–312. 10.1136/thoraxjnl-2018-211897 30244194 PMC6467246

[B74] Olejarz W. Kubiak-Tomaszewska G. Chrzanowska A. Lorenc T. (2020). Exosomes in angiogenesis and anti-angiogenic therapy in cancers. Int. J. Mol. Sci. 21, 5840. 10.3390/ijms21165840 32823989 PMC7461570

[B75] Oliveira-Rodríguez M. López-Cobo S. Reyburn H. T. Costa-García A. López-Martín S. Yáñez-Mó M. (2016). Development of a rapid lateral flow immunoassay test for detection of exosomes previously enriched from cell culture medium and body fluids. J. Extracell. Vesicles 5, 31803. 10.3402/jev.v5.31803 27527605 PMC4985618

[B76] Pan Y. Wong C. Y. Ma H. Tse R. T. Cheng C. K. Tan M. (2024). Quantitative comparison of the renal pelvic urine and bladder urine to examine modifications of the urine proteome by the lower urinary tract. Proteomics Clin. Appl. 18, e2300004. 10.1002/prca.202300004 37574260

[B77] Park J. Hwang M. Choi B. Jeong H. Jung J. H. Kim H. K. (2017). Exosome classification by pattern analysis of surface-enhanced raman spectroscopy data for lung cancer diagnosis. Anal. Chem. 89, 6695–6701. 10.1021/acs.analchem.7b00911 28541032

[B78] Pegtel D. M. Gould S. J. (2019). Exosomes. Annu. Rev. Biochem. 88, 487–514. 10.1146/annurev-biochem-013118-111902 31220978

[B79] Pengtao L. Kaiping B. Fei Y. Wei G. Xiangyu Z. Jie S. (2024). Plasma-derived exosomal hsa-miR-184 and hsa-mir-6766-3p as promising diagnostic biomarkers for early detection of children's cardiac surgery-associated acute kidney injury. Sci. Rep. 14, 22387. 10.1038/s41598-024-72737-w 39333590 PMC11436921

[B80] Pontis F. Roz L. Mensah M. Segale M. Moro M. Bertolini G. (2021). Circulating extracellular vesicles from individuals at high-risk of lung cancer induce pro-tumorigenic conversion of stromal cells through transfer of miR-126 and miR-320. J. Exp. Clin. Cancer Res. 40, 237. 10.1186/s13046-021-02040-3 34289890 PMC8293562

[B81] Rahimian S. Najafi H. Afzali B. Doroudian M. (2024). Extracellular vesicles and exosomes: novel insights and perspectives on lung cancer from early detection to targeted treatment. Biomedicines 12, 123. 10.3390/biomedicines12010123 38255228 PMC10813125

[B82] Rhim A. D. Mirek E. T. Aiello N. M. Maitra A. Bailey J. M. McAllister F. (2012). EMT and dissemination precede pancreatic tumor formation. Cell 148, 349–361. 10.1016/j.cell.2011.11.025 22265420 PMC3266542

[B83] Saman H. Raza A. Patil K. Uddin S. Crnogorac-Jurcevic T. (2022). Non-invasive biomarkers for early lung cancer detection. Cancers (Basel) 14, 5782. 10.3390/cancers14235782 36497263 PMC9739091

[B84] Sandfeld-Paulsen B. Jakobsen K. R. Bæk R. Folkersen B. H. Rasmussen T. R. Meldgaard P. (2016). Exosomal proteins as diagnostic biomarkers in lung cancer. J. Thorac. Oncol. 11, 1701–1710. 10.1016/j.jtho.2016.05.034 27343445

[B85] Shin H. Jeong H. Park J. Hong S. Choi Y. (2018). Correlation between cancerous exosomes and protein markers based on surface-enhanced raman spectroscopy (SERS) and principal component analysis (PCA). ACS Sens. 3, 2637–2643. 10.1021/acssensors.8b01047 30381940

[B86] Shin H. Oh S. Hong S. Kang M. Kang D. Ji Y. G. (2020). Early-stage lung cancer diagnosis by deep learning-based spectroscopic analysis of circulating exosomes. ACS Nano 14, 5435–5444. 10.1021/acsnano.9b09119 32286793

[B87] Silva S. Sousa J. C. Nogueira C. Feijo R. Neto F. M. Marinho L. C. (2024). Relationship between the expressions of DLL3, ASC1, TTF-1 and Ki-67: first steps of precision medicine at SCLC. Oncotarget 15, 750–763. 10.18632/oncotarget.28660 39392394 PMC11468345

[B88] Skallevold H. E. Vallenari E. M. Sapkota D. (2021). Salivary biomarkers in lung cancer. Mediat. Inflamm. 2021, 6019791. 10.1155/2021/6019791 34690552 PMC8528626

[B89] Skotland T. Llorente A. Sandvig K. (2023). Lipids in extracellular vesicles: what can be learned about membrane structure and function? Cold Spring Harb. Perspect. Biol. 15, a041415. 10.1101/cshperspect.a041415 37277192 PMC10411865

[B90] Song Y. Kelava L. Kiss I. (2023). MiRNAs in lung adenocarcinoma: role, diagnosis, prognosis, and therapy. Int. J. Mol. Sci. 24, 13302. 10.3390/ijms241713302 37686110 PMC10487838

[B91] Su X. Xie Y. Liu X. Chen M. Zheng C. Zhong H. (2023). Absolute quantification of serum exosomes in patients with an SERS-lateral flow strip biosensor for noninvasive clinical cancer diagnosis. ACS Appl. Mater Interfaces 15, 37130–37142. 10.1021/acsami.3c05039 37525365

[B92] Sun Y. Liu S. Qiao Z. Shang Z. Xia Z. Niu X. (2017). Systematic comparison of exosomal proteomes from human saliva and serum for the detection of lung cancer. Anal. Chim. Acta 982, 84–95. 10.1016/j.aca.2017.06.005 28734369

[B93] Sun Y. Huo C. Qiao Z. Shang Z. Uzzaman A. Liu S. (2018). Comparative proteomic analysis of exosomes and microvesicles in human saliva for lung cancer. J. Proteome Res. 17, 1101–1107. 10.1021/acs.jproteome.7b00770 29397740

[B94] Tao Y. Tang Y. Yang Z. Wu F. Wang L. Yang L. (2020). Exploration of serum exosomal LncRNA TBILA and AGAP2-AS1 as promising biomarkers for diagnosis of non-small cell lung cancer. Int. J. Biol. Sci. 16, 471–482. 10.7150/ijbs.39123 32015683 PMC6990900

[B95] Tu M. Wei F. Yang J. Wong D. (2015). Detection of exosomal biomarker by electric field-induced release and measurement (EFIRM). J. Vis. Exp. 23, 52439. 10.3791/52439 25650727 PMC4354565

[B96] Tzouvelekis A. Gomatou G. Bouros E. Trigidou R. Tzilas V. Bouros D. (2019). Common pathogenic mechanisms between idiopathic pulmonary fibrosis and lung cancer. Chest 156, 383–391. 10.1016/j.chest.2019.04.114 31125557

[B97] Verma N. Arora S. (2025). Navigating the global regulatory landscape for exosome-based therapeutics: challenges, strategies, and future directions. Pharmaceutics 17, 990. 10.3390/pharmaceutics17080990 40871013 PMC12389065

[B98] Verma N. Arora S. Singh A. K. Kumar A. (2025). Extracellular vesicle-associated miRNAs in cornea health and disease: diagnostic potential and therapeutic implications. Targets 3, 32. 10.3390/targets3040032

[B99] Vykoukal J. Sun N. Aguilar-Bonavides C. Katayama H. Tanaka I. Fahrmann J. F. (2017). Plasma-derived extracellular vesicle proteins as a source of biomarkers for lung adenocarcinoma. Oncotarget 8, 95466–95480. 10.18632/oncotarget.20748 29221141 PMC5707035

[B100] Wang K. Ru J. Zhang H. Chen J. Lin X. Lin Z. (2020). Melatonin enhances the therapeutic effect of plasma exosomes against cerebral ischemia-induced pyroptosis through the TLR4/NF-κB pathway. Front. Neurosci. 14, 848. 10.3389/fnins.2020.00848 33013286 PMC7461850

[B101] Wang F. Li X. Li M. Liu W. Lu L. Li Y. (2024). Ultra-short cell-free DNA fragments enhance cancer early detection in a multi-analyte blood test combining mutation, protein and fragmentomics. Clin. Chem. Lab. Med. 62, 168–177. 10.1515/cclm-2023-0541 37678194

[B102] Wang X. Song D. Zhu B. Jin Y. Cai C. Wang Z. (2024). Urinary exosomal mRNA as a biomarker for the diagnosis of bladder cancer. Anticancer Drugs 35, 362–370. 10.1097/CAD.0000000000001571 38385960 PMC10919263

[B103] Welsh J. A. Goberdhan D. C. I. O'Driscoll L. Buzas E. I. Blenkiron C. Bussolati B. (2024). Minimal information for studies of extracellular vesicles (MISEV2023): from basic to advanced approaches. J. Extracell. Vesicles 13, e12404. 10.1002/jev2.12404 38326288 PMC10850029

[B104] Wo Y. J. Gan A. S. P. Lim X. Tay I. S. Y. Lim S. Lim J. C. T. (2019). The roles of CD38 and CD157 in the solid tumor microenvironment and cancer immunotherapy. Cells 9, 26. 10.3390/cells9010026 31861847 PMC7017359

[B105] Wojtalewicz N. Vierbaum L. Kaufmann A. Schellenberg I. Holdenrieder S. (2023). Longitudinal evaluation of AFP and CEA external proficiency testing reveals need for method harmonization. Diagn. (Basel) 13, 2019. 10.3390/diagnostics13122019 37370914 PMC10296933

[B106] Xu H. Liao C. Zuo P. Liu Z. Ye B. C. (2018). Magnetic-based microfluidic device for On-Chip isolation and detection of tumor-derived exosomes. Anal. Chem. 90, 13451–13458. 10.1021/acs.analchem.8b03272 30234974

[B107] Xu X. Peng J. Wang N. Ocansey D. K. W. Zhang X. Mao F. (2024). hucMSC-Ex alleviates inflammatory bowel disease in mice by enhancing M2-type macrophage polarization *via* the METTL3-Slc37a2-YTHDF1 axis. Cell Biol. Toxicol. 40, 74. 10.1007/s10565-024-09921-1 39259386 PMC11390928

[B108] Xu F. Luo S. Lu P. Cai C. Li W. Li C. (2024). Composition, functions, and applications of exosomal membrane proteins. Front. Immunol. 15, 1408415. 10.3389/fimmu.2024.1408415 39148736 PMC11324478

[B109] Yang F. Yan Y. Yang Y. Hong X. Wang M. Yang Z. (2020). MiR-210 in exosomes derived from CAFs promotes non-small cell lung cancer migration and invasion through PTEN/PI3K/AKT pathway. Cell Signal 73, 109675. 10.1016/j.cellsig.2020.109675 32446904

[B110] Yasamineh S. Nikben N. Hamed A. M. Abdul Kareem R. Kadhim Al-Aridhy A. Hosseini Hooshiar M. (2024). Increasing the sensitivity and accuracy of detecting exosomes as biomarkers for cancer monitoring using optical nanobiosensors. Cancer Cell Int. 24, 189. 10.1186/s12935-024-03379-1 38816782 PMC11138050

[B111] Yuan G. Xie H. Wei T. Zhu D. Zhang C. Yang Y. (2021). Diagnostic potential of extracellular vesicle-associated microRNA-10b and tumor markers for lung adenocarcinoma. Oncol. Lett. 22, 614. 10.3892/ol.2021.12875 34257722 PMC8243083

[B112] Zabeti T. A. Norollahi S. E. Najafizadeh A. Babaei K. Bakhshalipour E. Vahidi S. (2024). Therapeutic combinations of exosomes alongside cancer stem cells (CSCs) and of CSC-derived exosomes (CSCEXs) in cancer therapy. Cancer Cell Int. 24, 334. 10.1186/s12935-024-03514-y 39369258 PMC11453077

[B113] Zandberg D. P. Hong C. S. Swartz A. Hsieh R. Anderson J. Ferris R. L. (2024). Small extracellular vesicles as biomarkers of response in recurrent/metastatic HNSCC patients treated with immunotherapy. BJC Rep. 2, 70. 10.1038/s44276-024-00096-0 39281316 PMC11390474

[B114] Zang X. Gu J. Zhang J. Shi H. Hou S. Xu X. (2020). Exosome-transmitted lncRNA UFC1 promotes non-small-cell lung cancer progression by EZH2-mediated epigenetic silencing of PTEN expression. Cell Death Dis. 11, 215. 10.1038/s41419-020-2409-0 32242003 PMC7118073

[B115] Zeng Z. Li Y. Pan Y. Lan X. Song F. Sun J. (2018). Cancer-derived exosomal miR-25-3p promotes pre-metastatic niche formation by inducing vascular permeability and angiogenesis. Nat. Commun. 9, 5395. 10.1038/s41467-018-07810-w 30568162 PMC6300604

[B116] Zhang H. Lu H. Xiang L. Bullen J. W. Zhang C. Samanta D. (2015). HIF-1 regulates CD47 expression in breast cancer cells to promote evasion of phagocytosis and maintenance of cancer stem cells. Proc. Natl. Acad. Sci. U. S. A. 112, E6215–E6223. 10.1073/pnas.1520032112 26512116 PMC4653179

[B117] Zhang N. Nan A. Chen L. Li X. Jia Y. Qiu M. (2020). Circular RNA circSATB2 promotes progression of non-small cell lung cancer cells. Mol. Cancer 19, 101. 10.1186/s12943-020-01221-6 32493389 PMC7268724

[B118] Zhang W. Tian Z. Yang S. Rich J. Zhao S. Klingeborn M. (2021). Electrochemical micro-aptasensors for exosome detection based on hybridization chain reaction amplification. Microsyst. Nanoeng. 7, 63. 10.1038/s41378-021-00293-8 34567775 PMC8433316

[B119] Zhou P. Lu F. Wang J. Wang K. Liu B. Li N. (2020). A portable point-of-care testing system to diagnose lung cancer through the detection of exosomal miRNA in urine and saliva. Chem. Commun. (Camb) 56, 8968–8971. 10.1039/d0cc03180a 32638761

[B120] Zhou X. Jia Y. Mao C. Liu S. (2023). Small extracellular vesicles: non-negligible vesicles in tumor progression, diagnosis, and therapy. Cancer Lett. 1, 216481. 10.1016/j.canlet.2023.216481 37972701

[B121] Zhu X. Xie M. Cui Y. (2025). Long non-coding RNA SNHG16 promotes tumor progression and cisplatin resistance in esophageal squamous cell carcinoma *via* miR-497-5p/HK2 axis. J. Cardiothorac. Surg. 20, 298. 10.1186/s13019-025-03528-1 40660293 PMC12257804

[B122] Zhuang T. Wang S. Yu X. He X. Guo H. Ou C. (2024). Current status and future perspectives of platelet-derived extracellular vesicles in cancer diagnosis and treatment. Biomark. Res. 12, 88. 10.1186/s40364-024-00639-0 39183323 PMC11346179

